# Changing from the CKD-EPI to the EKFC creatinine equation to estimate glomerular filtration rate in adults in a Northern European health system

**DOI:** 10.1093/ndt/gfaf148

**Published:** 2025-08-11

**Authors:** William A Russel, Edouard L Fu, Antoine Creon, Anne-Laure Faucon, Aurora Caldinelli, Pim Bouwmans, Lesley A Inker, Pierre Delanaye, Hans Pottel, Alberto Ortiz, Andrew S Levey, Juan J Carrero

**Affiliations:** Department of Medical Epidemiology and Biostatistics, Karolinska Institutet, Stockholm, Sweden; Department of Medical Epidemiology and Biostatistics, Karolinska Institutet, Stockholm, Sweden; Department of Clinical Epidemiology, Leiden University Medical Center, Leiden, The Netherlands; Department of Medical Epidemiology and Biostatistics, Karolinska Institutet, Stockholm, Sweden; Department of Medical Epidemiology and Biostatistics, Karolinska Institutet, Stockholm, Sweden; Department of Medical Epidemiology and Biostatistics, Karolinska Institutet, Stockholm, Sweden; Department of Medical Epidemiology and Biostatistics, Karolinska Institutet, Stockholm, Sweden; Cardiovascular Research Institute Maastricht, Maastricht University, Maastricht, The Netherlands; Division of Nephrology, Department of Internal Medicine, Tufts Medical Center, Boston, MA, USA; Department of Nephrology-Dialysis-Transplantation, University of Liège, CHU Sart Tilman, Liège, Belgium; Department of Nephrology-Dialysis-Apheresis, Hôpital Universitaire Carémeau, Université de Montpellier, Nîmes, France; Department of Public Health and Primary Care, KULeuven Campus Kulak Kortrijk, Leuven, Belgium; Department of Nephrology and Hypertension, IIS-Fundacion Jimenez Diaz UAM, Madrid, Spain; Division of Nephrology, Department of Internal Medicine, Tufts Medical Center, Boston, MA, USA; Department of Medical Epidemiology and Biostatistics, Karolinska Institutet, Stockholm, Sweden; Division of Nephrology, Department of Clinical Sciences, Karolinska Institutet, Danderyd Hospital, Stockholm, Sweden

**Keywords:** CKD-EPI, creatinine, EKFC, epidemiology, glomerular filtration rate

## Abstract

**Background:**

The European Kidney Function Consortium (EKFC) 2021 equation to estimate GFR performs as well or better than the CKD-EPI 2009 equation in predominantly White adult European populations, with less bias and greater accuracy against measured GFR. This study explores how changing from the CKD-EPI to the EKFC equation in a large European health system may affect disease distribution, prognosis, and clinical decisions.

**Methods:**

We studied >1.7 million adults in Stockholm undergoing routine care during 2006–2021. We compared eGFR values and reclassification across KDIGO GFR categories when changing from the CKD-EPI to EKFC equation and examined associations of eGFR and reclassification with risk for kidney failure with replacement therapy (KFRT), mortality, and major adverse cardiovascular events (MACE) using Cox models. We also modeled the impact of eGFR equation change on clinical decisions such as nephrology referral or medication eligibility/contraindication.

**Results:**

EKFC yielded modestly lower eGFR values than CKD-EPI by a median (IQR) of −4.9 (−8.3 to −2.2) ml/min/1.73 m². As a result, CKD G3–G5 prevalence rose from 4.5% to 6.2%. Both equations strongly predicted KFRT, mortality, and MACE. Participants reclassified to lower eGFR categories were older; after adjustment for age, participants had similar risks of mortality and MACE to those not reclassified and a lower risk of KFRT. Changing to the EKFC equation would impact clinical decisions at low eGFR thresholds, such as nephrology referrals (22% higher), eligibility for SGLT2 is (39% higher), or contraindication for spironolactone in heart failure (26% higher).

**Conclusions:**

Adopting the EKFC equation in this Northern European health system would modestly lower eGFR estimates, increasing the prevalence of moderate/severe CKD and affecting clinical classification and decisions. eGFR by both equations strongly predicted outcomes, but individuals reclassified to a lower eGFR category by EKFC did not have consistent associations across outcomes.

## Graphical Abstract

**Graphical Abstract:**
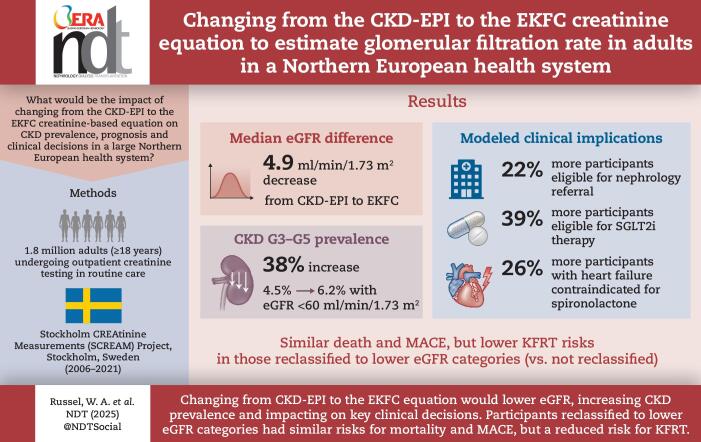

KEY LEARNING POINTS **What was known:**

- The Chronic Kidney Disease Epidemiology Collaboration (CKD-EPI) 2009 creatinine-based equation is recommended for routine estimation of glomerular filtration rate (eGFR) in Europe.
- However, recent studies in select, predominantly White European populations suggest that the European Kidney Function Consortium (EKFC) 2021 equation may have similar or slightly improved bias and precision against measured GFR.
- Proposals have emerged for European health systems to adopt the EKFC equation for routine use, but the impact of changing from the CKD-EPI to EKFC equation has not been shown outside research cohorts.

**This study adds:**

- In >1.7 million individuals accessing healthcare in Stockholm, we found that changing from the CKD-EPI to EKFC equation would lower eGFR by a modest amount, which may affect many important clinical classifications across eGFR thresholds that are used to guide care and inform treatment decisions.
- Both equations were strong predictors of kidney, cardiovascular, and mortality outcomes.
- Participants reclassified to lower eGFR categories had a greater risk of mortality and major adverse cardiovascular events than those not reclassified, explained by their older age, but a lower risk of kidney failure with replacement therapy.

**Potential impact:**

- Changing from the CKD-EPI to EKFC equation would have major effects on classification of eGFR categories in adults in European health systems, but factors other than creatinine-based eGFR affect treatment decisions, and identification of individuals at risk for kidney failure was not improved.
- Awareness of these implications is important for healthcare professionals, researchers, and policy makers when evaluating the change of using EKFC instead of CKD-EPI to estimate GFR.

## INTRODUCTION

Estimated glomerular filtration rate (eGFR) is the primary clinical tool for assessing kidney function. It informs the diagnosis and

staging of chronic kidney disease (CKD), guides nephrology referral, and determines medication eligibility and dosing for medications cleared by the kidneys [1]. eGFR is routinely calculated using serum creatinine (eGFR_cr_), and this value is used to initiate clinical responses based on whether it falls above or below established GFR thresholds. Clinical decisions about intervention are then guided by a more holistic assessment that includes the underlying cause of CKD and the urinary albumin-to-creatinine ratio (uACR), and may incorporate eGFR based on serum cystatin C, alone or with creatinine (eGFR_cys_ or eGFR_cr-cys_) when non-GFR determinants of creatinine are suspected [2].

To promote uniformity in GFR estimation, the KDIGO 2012 guidelines [3] recommended using the Chronic Kidney Disease Epidemiology Collaboration (CKD-EPI [4]) 2009 eGFR_cr_ equation, unless another eGFR_cr_ equation was more accurate. The recent 2024 KDIGO guideline update [2] acknowledges that the accuracies of eGFR equations vary across regions with different population characteristics and now encourages the use of a validated eGFR equation that best fits the region in which it is to be used. This change reflects, in part, the adoption of the race-free CKD-EPI 2021 equation in the USA to advance health equity, while European health systems have retained the CKD-EPI 2009 equation with the race coefficient set to non-Black, as it is more accurate in predominantly White populations [5].

In response to these evolving regional practices, interest has grown in the European Kidney Function Consortium (EKFC) 2021 eGFR_cr_ equation [6]. In general, both the CKD-EPI 2009 and EKFC 2021 equations perform well in predominantly White European populations, with relatively small differences in bias and accuracy when compared to measured GFR. In studies using similar methods to measure GFR as were used in the development of the EKFC equation, the EKFC equation tends to yield slightly lower eGFR values than CKD-EPI, which may manifest as reduced positive bias or a modest underestimation of mGFR, depending on the study population [7, 8]. Since select studies in European populations have shown potential improvements using EKFC instead of CKD-EPI, proposals have emerged for European health systems to change from the CKD-EPI to EKFC equation for routine eGFR_cr_ determination [9].

Since the EKFC equation reduces bias in GFR estimation, it is important for European health systems to understand how changing from the CKD-EPI to EKFC equation might alter regional distributions of eGFR and shift the classification of individuals across clinically actionable thresholds. Whether this change would lead to improved classification and management of individuals at risk for kidney-related outcomes is a key part of this consideration. While these questions have been explored in previous work [10], these insights emanate from a smaller, clinically selected cohort, which may not capture the broader implications of this change at the population level.

This study evaluates the clinical implications of changing from the CKD-EPI to EKFC eGFR_cr_ equation in a predominantly White Northern European healthcare system consisting of >1.7 million individuals. To assess the consequences of this change, we investigated: (i) differences in eGFR and their relation to participant characteristics, (ii) associations of equation eGFR and reclassification with risks of mortality, cardiovascular events, and kidney failure, and (iii) potential implications on nephrologist referral and treatment decisions for common medications.

## MATERIALS AND METHODS

### Data source

The study population consisted of participants in the Stockholm CREAtinine Measurements (SCREAM) project, a healthcare utilization cohort including residents in the Stockholm region, Sweden [11]. The region of Stockholm contains 20%–25% of the population of Sweden, and has one single healthcare provider that provides universal and tax-funded healthcare. The study was approved by the Regional Ethical Review Board in Stockholm (2017/793–31).

### Study population

We included all adult (≥18 years) participants who had at least one outpatient creatinine measurement taken during 2006–2021 and no previous history of kidney failure with replacement therapy (KFRT). The date of the first creatinine measurement fitting these criteria was the index date of the study. Patients were followed from index date to the first occurrence of a study outcome, death, or end of follow-up (December 2021).

### CKD-EPI 2009 and EKFC 2021 creatinine equations

At the index date, we calculated eGFR with the CKD-EPI 2009 and EKFC 2021 creatinine equations, hereafter referred to as the CKD-EPI and EKFC equations [4, 6]. Creatinine was measured in serum or plasma, with either an enzymatic or corrected Jaffe method (alkaline picrate reaction); both methods are traceable to isotope dilution mass spectroscopy standards. For the CKD-EPI equation, eGFR was calculated using the non-Black coefficient [12]. For the EKFC equation, we chose the *Q* values derived from a White Swedish population (Supplementary Methods). Race was not available, because Sweden does not allow data collection on ethnicity to prevent discrimination.

### Statistical analyses

#### Changes in eGFR with the EKFC versus CKD-EPI equation

First, we assessed the median eGFR of both equations across the spectrum of age with spline plots. We evaluated how eGFR differences varied by CKD-EPI eGFR level, age, and sex by plotting eGFR difference against the eGFR range, using kernel density estimation to depict difference trends among age subgroups.

To assess reclassification, we cross-tabulated the proportion of individuals with eGFR using the CKD-EPI and EKFC equations classified to eGFR categories (≥90, 60–89, 45–59, 30–44, 15–29, <15 ml/min/1.73 m^2^, which correspond to GFR categories G1, G2, G3a, G3b, G4, and G5, respectively) used for the current definition and classification of CKD [2]. We calculated the proportion of individuals in each CKD-EPI eGFR category who were reclassified to a higher or lower GFR category by the EKFC. We also compared characteristics between reclassified and non-reclassified individuals. Covariates are defined in Supplementary Table S1, with no missing values.

#### Association between eGFR and study outcomes

We described the association between eGFR by both equations and the outcomes KFRT, all-cause death, and major adverse cardiovascular events (MACE) by fitting a cause-specific Cox model, modeling eGFR with a restricted cubic spline with five knots placed on the 5th, 27.5th, 50th, 72.5th, and 95th percentile of the data. An eGFR of 95 ml/min/1.73 m^2^ was taken as the reference value [13], even though it has been questioned previously. In the primary analysis, we present hazard ratios adjusted for age and sex, as well as comorbidities and medication use to align with prior investigations and avoid confusion for a clinical audience. We first describe the unadjusted hazard ratios to reflect the prognostic value of the initial eGFR used for CKD staging. Since age and sex are components of both eGFR equations, and their weights differ between CKD-EPI and EKFC, adjusting for these variables could obscure the effects of coefficient differences on risk associations. In a sensitivity analysis, we describe these associations using a reference value of 85 ml/min/1.73 m^2^ to test the robustness of these associations with health outcomes. KFRT was defined as a composite outcome including initiation of maintenance dialysis or kidney transplantation, without considering decisions on conservative care. MACE was defined as a composite of cardiovascular death, non-fatal myocardial infarction, or non-fatal stroke (Supplementary Table S2). Individuals were followed until the first occurrence of the study outcome, death, emigration from the region, or end of follow-up (31 December 2021). For the outcomes of KFRT, death, and MACE, we accounted for the competing risk of death by setting the follow-up time to the administrative censoring time [14].

We compared the risk of outcomes between reclassified and non-reclassified individuals in the same eGFR category (e.g. we compared the risk of KFRT among those who were reclassified from G3b to G4 with those who remained in G3b). Since reclassified individuals are often closer to the GFR threshold than non-reclassified individuals, this may explain differences in health outcomes. To account for this, we adjusted for their CKD-EPI eGFR, thus comparing reclassified participants to non-reclassified participants given the same CKD-EPI eGFR level. Finally, we further adjusted for age and sex to evaluate the degree to which these characteristics explained differences in risk. Our initial analyses explored reclassification across all GFR categories (G1–G5), but because the number of reclassified individuals in some G categories was low, we repeated the analyses merging the categories G3a–G5 into one category. Finally, we calculated the net reclassification index (NRI, Supplementary Methods).

#### Clinical implications

We explored selected clinical implications of changing from the CKD-EPI to EKFC equation. The criterion considered for nephrologist referral was reaching an eGFR <30 ml/min/1.73 m^2^ [2]. The criterion for eligibility for SGLT2i was to have an eGFR between 20 and <60 ml/min/1.73 m² [15]. Conversely, reaching an eGFR <20 ml/min/1.73 m² was a criterion for being non-eligible for SGLT2i [ALSO NHS]. Among people with diabetes, we assessed how many individuals would become eligible for GLP-1 treatment (<60 ml/min/1.73 m² [16]) or non-eligible for metformin (<30 ml/min/1.73 m² [17]). Among people with heart failure, we estimated how many individuals would become non-eligible for spironolactone (<30 ml/min/1.73 m² [18]). Among people with atrial fibrillation, we assessed how many individuals would become non-eligible for apixaban (<15 ml/min/1.73 m²) or require a dose reduction (if eGFR 15 to <50 m /min/1.73 m²) [19].

#### Sensitivity analysis

Prior studies show that small differences in eGFR between equations (up to ±5 ml/min/1.73 m^2^) can be due to differences in measurement methods for mGFR or filtration marker concentrations, rather than clinically meaningful differences in accuracy of equations [8, 20]. Thus, we repeated our analyses considering only reclassification because of a clinically meaningful difference (>±5 ml/min/1.73 m² between CKD-EPI and EKFC eGFR values). We note that a fixed absolute difference may carry different clinical significance at a lower eGFR where measurement variability is reduced than at higher eGFR where variability is greater [21]. In this sensitivity analysis, participants with a change in GFR categories but an eGFR difference of 5 ml/min/1.73 m^2^ or less was considered as non-reclassified.

## RESULTS

We included total 1 784 831 participants (Supplementary Fig. S1) with mean age 46 years, 53% women, and mean creatinine of 73 μmol/l (Supplementary Table S3). The most common comorbid conditions were hypertension, diabetes, and arrythmia, and the most used medications were non-steroidal anti-inflammatory drugs, renin–angiotensin system inhibitors, and beta-blockers. Individuals classified in lower eGFR categories were older, more often women, had more comorbid conditions, and took more medications with either equation (Supplementary Tables S3 and S4).

### Differences in eGFR

**Figure 1:**
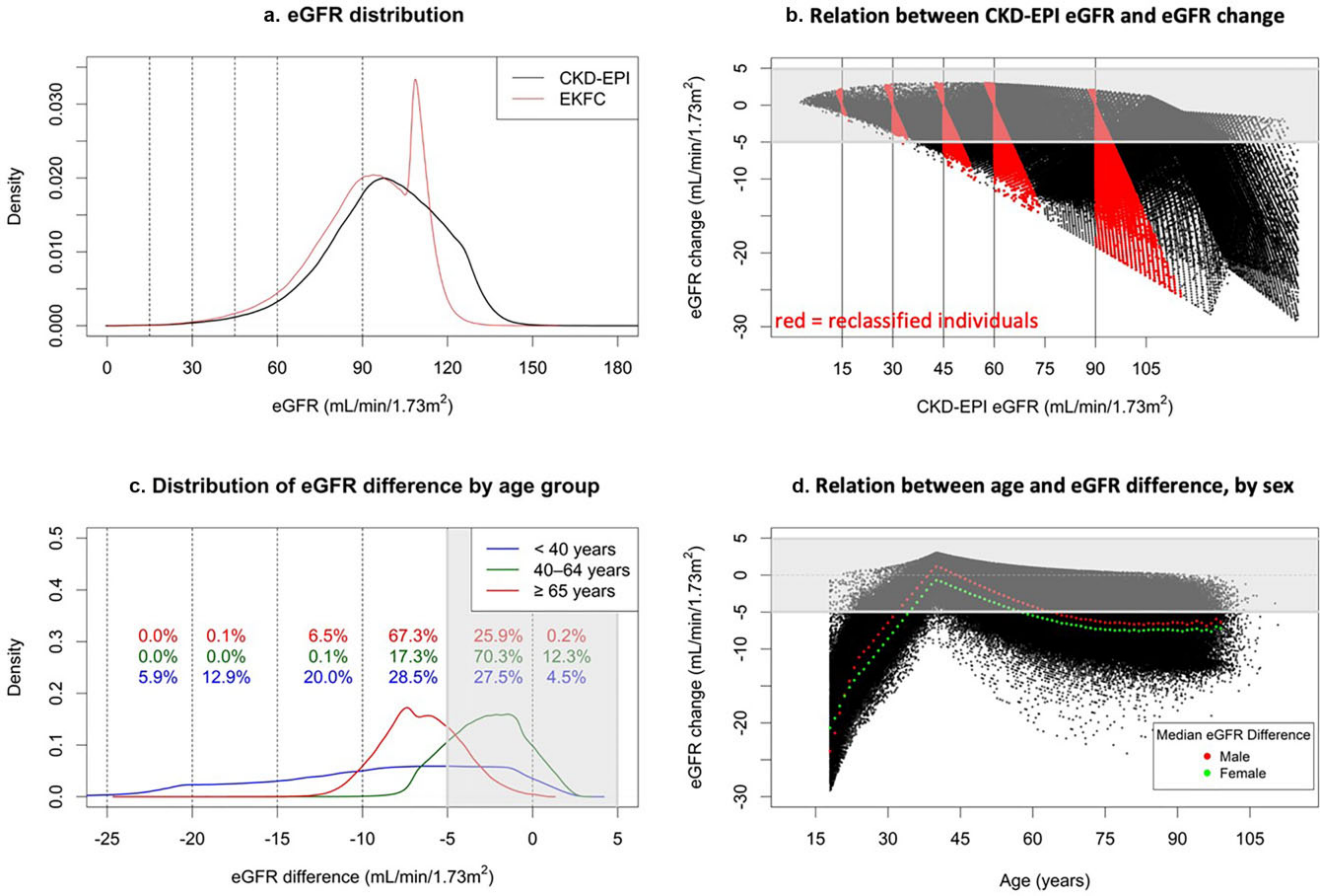
(**a**) eGFR distributions for CKD-EPI and EKFC equations. (**b**) Relation between CKD-EPI eGFR and eGFR difference when changing to the EKFC equation. Each dot represents an individual, red dots denote upward and downward reclassified individuals, and vertical lines depict KDIGO eGFR thresholds. (**c**) Distribution of eGFR difference when changing from the CKD-EPI to EKFC equation, separately for those <40, 40 to 64, and ≥65 years. Distributions are based on kernel density estimation, and numbers depict the proportion of participants in each age subgroup within categories of eGFR difference in 5 ml/min/1.73 m^2^ increments from −25 to +5 ml/min/1.73 m^2^. (**d**) Relation between age and difference in eGFR when changing from the CKD-EPI to EKFC equation, with mean difference shown separately for both sexes. Each black dot represents an individual, with red and green dots showing the mean difference in eGFR between the CKD-EPI and EKFC equation for males and females, respectively, by 1-year age strata. Gray shaded areas in (b)–(d) represent small magnitude differences ≤5 ml/min/1.73 m^2^ between CKD-EPI and EKFC eGFR.

eGFR with EKFC was lower than CKD-EPI, with an overall median difference of −4.9 (IQR −8.3, −2.2) ml/min/1.73 m^2^ (Fig. 1). The sharp increase in individuals with an eGFR 110–115 ml/min/1.73 m^2^ observed in the EKFC density curve (Fig. 1a) is likely due to the structure of the EKFC equation, which applies no age adjustment for individuals under 40 years, resulting in similar median eGFR values for these participants (Supplementary Fig. S2). eGFR differences were larger at higher eGFR (Fig. 1b) and at older and younger ages (Fig. 1c and d), surpassing our predefined threshold for clinically meaningful difference (>5 ml/min/1.73 m^2^). Median differences in eGFR for older (≥65 yrs), middle-aged (40 to 64 yrs) and younger (<40 yrs) participants were −6.6 (IQR −8.1, −4.9), −2.6 (−4.3, −1.0), and −8.1 (−13.3, −3.8) ml/min/1.73 m^2^, respectively. Females had larger eGFR differences than males (Fig. 1d). eGFR differences were consistent across subgroups (Supplementary Table S5, Supplementary Fig. S3).

### Association between eGFR and study outcomes

**Figure 2:**
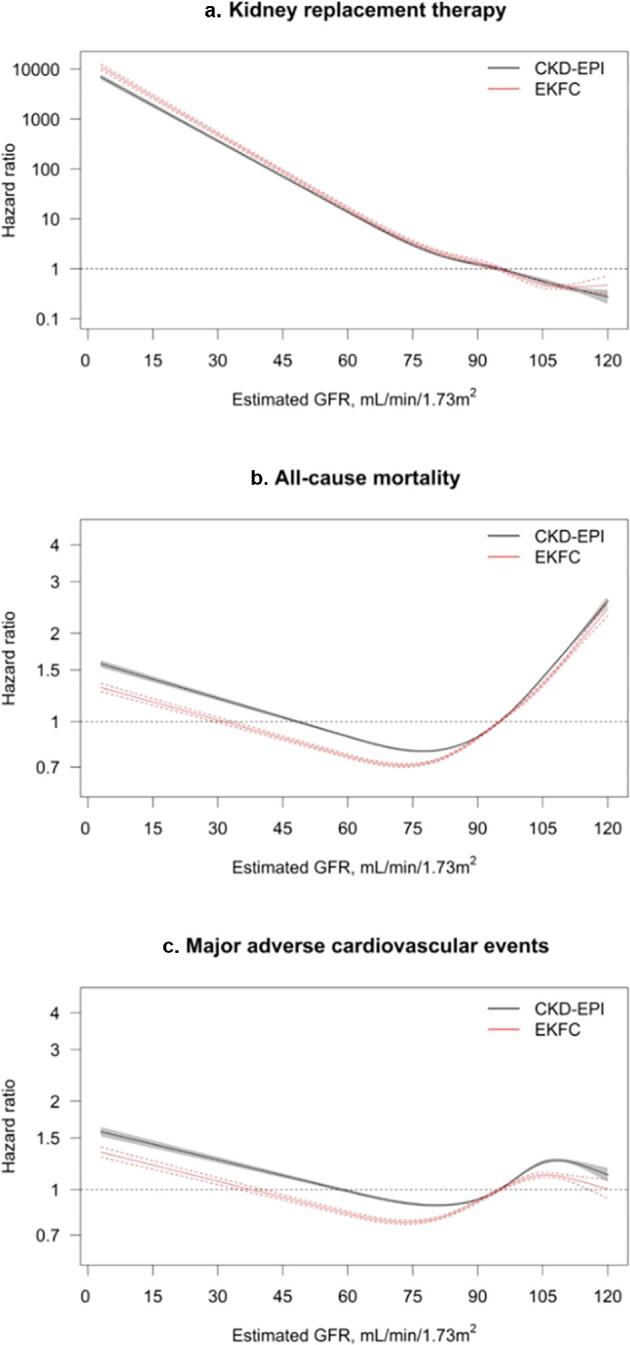
Hazard ratios for the association between eGFR with CKD-EPI and EKFC equations and KFRT (**a**), all-cause mortality (**b**), and MACE (**c**) adjusted for age, sex, comorbidities, and medication use. An eGFR of 95 ml/min/1.73 m^2^ was taken as the reference value. Age was modeled linearly in the Cox model. Hazard ratios were adjusted for comorbidities (hypertension, myocardial infarction, ischemic heart disease, heart failure, stroke, cerebrovascular disease, arrhythmia, peripheral vascular disease, diabetes mellitus, cancer, chronic obstructive disease, and liver disease) and medication use (β-blockers, calcium channel blockers, diuretics, renin–angiotensin system inhibitors, lipid-lowering drugs, and NSAIDs).

**Figure 3:**
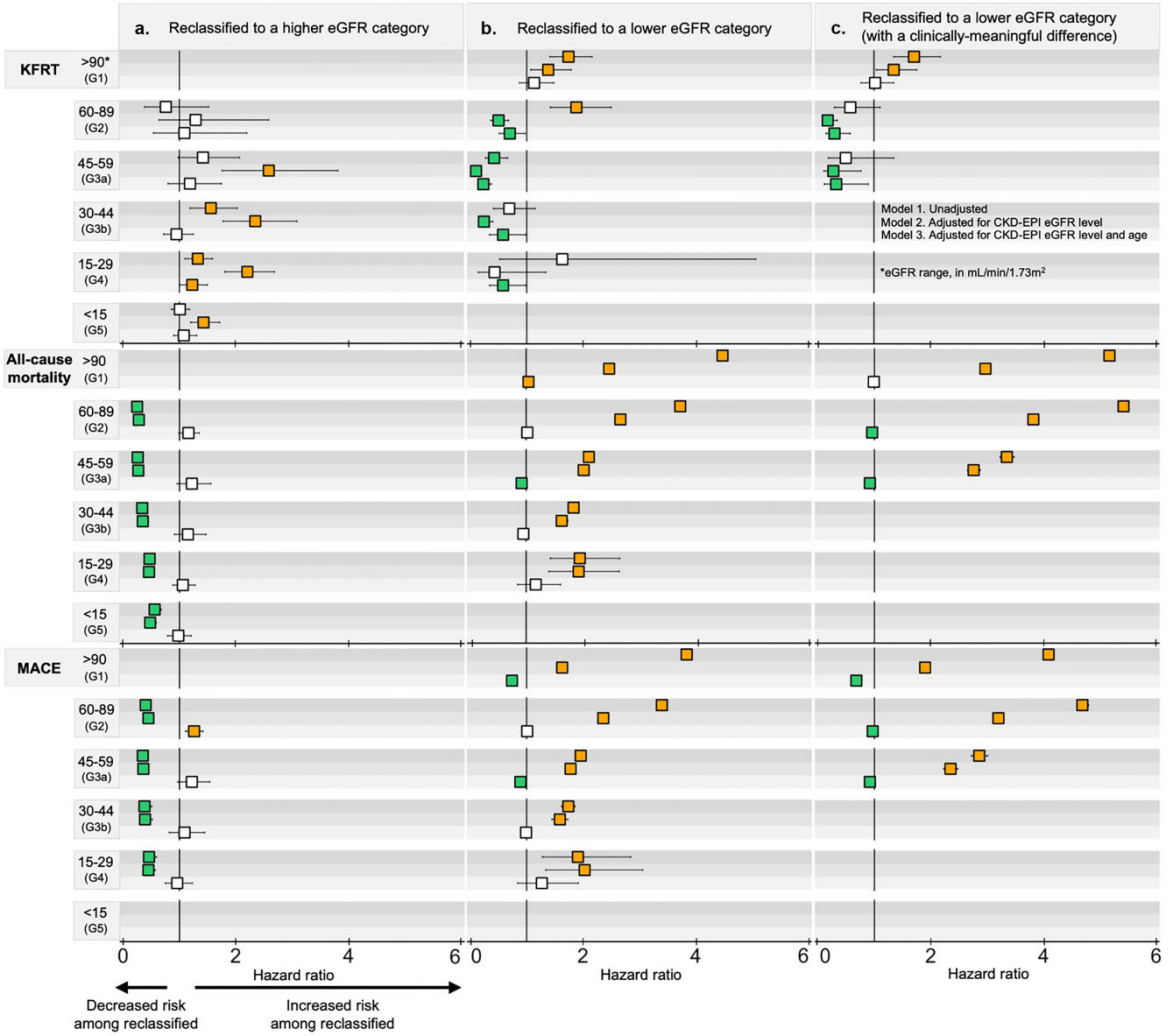
Hazard ratios comparing reclassified and non-reclassified participants for the risk of KFRT, all-cause mortality, and MACE. Comparisons include participants reclassified to a higher eGFR category by EKFC versus non-reclassified individuals who remained in the same eGFR category designated by CKD-EPI (**a**), participants reclassified to a lower eGFR category versus those not reclassified (**b**), and those reclassified downward with a clinically meaningful eGFR difference of >5 ml/min/1.73 m^2^ versus non-reclassified participants (**c**). Within each eGFR category, results are shown for three models: unadjusted, adjusted for CKD-EPI eGFR, and further adjusted for age (from top to bottom). Cell colors indicate the direction of risk based on the hazard ratio and its 95% confidence interval: white for no statistically significant difference, orange for statistically significant (*P* < .05) increased risk, and green for statistically significant reduced risk.

During median 10.9 (5.9–14.2) years, 233 404 participants died, 143 928 experienced MACE, and 3039 started KFRT. eGFR with either CKD-EPI or EKFC equations was similarly associated with all outcomes, with hazard ratios rising inversely to eGFR (Fig. 2, Supplementary Fig. S4). Similar findings were observed after adjustment, but the relationships between eGFR with all-cause mortality and MACE were attenuated and became U-shaped instead of linear on the log scale (Fig. 3, Supplementary Fig. S5). These trends were consistent when using a reference eGFR value of 85 ml/min/1.73 m^2^ (Supplementary Fig. S6–S8).

### Reclassification proportions

Here, 203 758 participants (11%) were reclassified to a lower eGFR category with the EKFC equation, and 3777 participants (<1%) were reclassified to a higher eGFR category (Table 1a). Consequently, the prevalence of categories G3–G5 increased from 4.5% to 6.2%. When restricting the analysis to reclassifications exceeding our threshold for clinically meaningful difference, only individuals in G1–G3 were reclassified; a total of 147 956 participants (8% of the cohort) were reclassified to a lower eGFR category and no participants were reclassified to a higher eGFR category (Table 1b).

**Table 1:** Reclassification across eGFR categories if changing from the 2009 CKD-EPI equation to the 2021 EKFC equation. (**a**) All reclassifications are shown overall. (**b**) Only reclassifications with a clinically meaningful eGFR difference from CKD-EPI to EKFC of >5 ml/min/1.73 m^2^ are shown. Bold cells represent the participants that are classified in the same eGFR category by both equations, while cells to the right of the bold cells represent those reclassified to a lower eGFR category by EKFC 2021 and cells to the left represent those reclassified to a higher eGFR category by CKD-EPI 2009.

	eGFR category with the CKD-EPI equation, ml/min/1.73 m^2^ (G category)						
eGFR category with the CKD-EPI equation, ml/min/1.73 m^2^ (G category)	>90 (G1)	60–89 (G2)	45–59 (G3a)	30–44 (G3b)	15–29 (G4)	<15 (G5)	Total
(a) All reclassifications occurring across eGFR category thresholds							
>90 (G1)	**1 033 879 (86.5%)**	161 643 (13.5%)	0	0	0	0	1 195 522 (67%)
60–89 (G2)	3 162 (0.6%)	**475 399 (93.3%)**	31 248 (6.1%)	0	0	0	509 809 (28.6%)
45–59 (G3a)	0	364 (0.7%)	**44 318 (82%)**	9 335 (17.3%)	0	0	54 017 (3%)
30–44 (G3b)	0	0	99 (0.5%)	**17 512 (91.7%)**	1 490 (7.8%)	0	19 101 (1.1%)
15–29 (G4)	0	0	0	81 (1.5%)	**5 313 (97.7%)**	42 (0.8%)	5 436 (0.3%)
<15 (G5)	0	0	0	0	71 (7.5%)	**871 (92.5%)**	942 (0.1%)
Total	1 037 041 (58.1%)	637 406 (35.7%)	75 665 (4.2%)	26 928 (1.5%)	6 874 (0.4%)	913 (0.1%)	**1 784 827** 3 777 **| 1 577 292 |** 203 758
**(b) Reclassified with clinically meaningful eGFR difference**							
>90 (G1)	**1 071 463 (89.6%)**	124 059 (10.4%)	0	0	0	0	1 195 522 (67%)
60–89 (G2)	0	**488 986 (95.9%)**	20 825 (4.1%)	0	0	0	509 811 (28.6%)
45–59 (G3a)	0	0	**50 945 (94.3%)**	3 072 (5.7%)	0	0	54 017 (3%)
30–44 (G3b)	0	0	0	**19 102 (100%)**	1 (0%)	0	19 103 (1.1%)
15–29 (G4)	0	0	0	0	**5 436 (100%)**	0	5 436 (0.3%)
<15 (G5)	0	0	0	0	0	**942 (100%)**	942 (0.1%)
Total	1 071 463 (60.0%)	613 045 (34.3%)	71 770 (4.0%)	22 174 (1.2%)	5 437 (0.3%)	942 (0.1%)	**1 784 827** 0 **| 1 636 870 |** 147 957

Participants reclassified to a higher eGFR category were younger, mostly male, had fewer comorbid conditions, and used fewer medications (Table 2). Participants reclassified to a lower eGFR category were older, mostly women, had more comorbid conditions, and used more medications. Reclassified individuals had eGFR values closer to eGFR category thresholds than non-reclassified participants (Supplementary Table S6). These trends were consistent among participants in categories G3–G5 (Supplementary Table S7), and adjustment for age revealed that comorbid conditions were not different between reclassified and non-reclassified groups (Supplementary Table S8). When restricted to reclassifications designated as clinically meaningful, participants continued to be older with more comorbid conditions (Table 2).

**Table 2:** Baseline characteristics of reclassified and non-reclassified individuals when changing from the CKD-EPI 2009 to EKFC 2021 equation for individuals classified to CKD G3-5 with CKD-EPI 2009. Sample sizes are expressed as percentages out of the total number of individuals in each population subgroup (RASi, renin–angiotensin system inhibitor; NSAIDs, non-steroidal anti-inflammatory drugs).

	Total individuals (*n* = 1 784 831)			Total individuals (*n* = 1 784 831)	
Characteristic	Reclassified to higher eGFR category (*n* = 3 777)	Not reclassified (*n* = 1 636 874)	Reclassified to lower eGFR category (*n* = 203 758)	Not reclassified (*n* = 1 577 296)	Reclassified to lower eGFR category with meaningful eGFR difference (*n* = 147 957)
Mean age (SD), y	42 (4)	45 (18)	56 (21)	45 (18)	56 (23)
<20	0	5	8	5	11
20–39	27	39	18	40	20
40–59	72	35	15	35	4
60–69	0.8	10	35	10	41
70–79	0.1	7	12	7	12
≥80	0	4	12	4	13
Female sex, %	9	52	57	53	61
Mean plasma creatinine (SD), μmol/l	102 (43)	73 (21)	75 (17)	73 (20)	71 (14)
eGFR with the CKD-EPI equation, ml/min/1.73 m^2^	83 (17)	98 (21)	87 (15)	99 (21)	90 (13)
eGFR with the EKFC equation, ml/min/1.73 m^2^	84 (17)	93 (19)	80 (14)	93 (18)	81 (12)
Education, %					
Compulsory school	10	16	23	16	24
Secondary school	38	39	40	39	40
University	53	45	37	45	36
Medical history, %					
Hypertension	8	11	21	11	20
Myocardial infarction	0.6	2	3	2	3
Other ischemic heart disease	0.7	3	7	3	6
Heart failure	0.9	2	4	2	4
Stroke	1	2	4	2	4
Other cerebrovascular disease	0.7	2	3	2	3
Arrhythmia	2	4	7	4	7
Peripheral vascular disease	0.5	0.8	2	0.8	2
Diabetes	3	5	7	5	7
Cancer	1	2	4	2	4
Chronic obstructive pulmonary disease	0.3	1	3	1	3
Liver disease	1	1	1	1	1
Concomitant medications, %					
Beta blocker	6	9	16	9	15
Calcium channel blocker	4	4	8	4	8
Diuretic	3	6	12	6	11
RASi	7	8	14	8	13
Lipid-lowering drug	3	6	12	6	11
NSAIDs	12	12	14	12	13
Calendar year, %					
2007–2010	51	58	66	58	65
2011–2014	20	18	14	19	15
2015–2019	18	14	12	14	13
2020–2021	11	10	7	10	8

We assessed the risk for reclassified participants compared to those not reclassified (Fig. 3, estimates and event counts given in Supplementary Table S9). For participants reclassified to a higher eGFR category, unadjusted models showed lower risks for all-cause mortality and MACE for all eGFR categories, but variable risk for KFRT by eGFR category (Fig. 3a). Adjustment for CKD-EPI eGFR level and age generally attenuated associations (Fig. 3a). For participants reclassified to a lower eGFR category, unadjusted models showed higher risks of all-cause mortality and MACE for all eGFR categories, but variable risk for KFRT by eGFR category (Fig. 3b). Adjustment for eGFR and age brought most observed risk associations to the null, except for associations with KFRT, which were lower for G2–G4 (Fig. 3b). These trends for upward and downward reclassified participants remained when combining categories G3–G5 into a single category (Supplementary Table S10). When we considered only designated meaningful reclassifications, we still observed that individuals reclassified from categories G2 or G3a to a lower eGFR category were at a lower risk for KFRT (Fig. 3c).

Overall NRI was negative for KFRT but positive for all-cause mortality and MACE (Table 3). For KFRT, the event and non-event NRI were 1.9% (95% CI 1.0, 3.3%) and −11.2% (−11.3, −11.2%), respectively, meaning that 1.9% of participants who experienced KFRT were correctly reclassified to a higher risk eGFR category by the EKFC equation, but 11.2% of participants who did not experience KFRT were incorrectly reclassified to a higher eGFR risk category (Table 3a). Event NRI were greater for all-cause mortality and MACE [22.1% (95%CI, 21.9, 22.3%) and 20.9% (20.7, 21.1%), respectively], while non-event NRI remained similar across these outcomes (−9.8% (−9.9, −9.8%) and −10.4% (-10.5, −10.4%), respectively). When limiting the analysis to reclassified individuals whose difference was classified as clinically meaningful eGFR, the overall, event, and non-event NRI for study outcomes were not substantially different (Table 3b, Supplementary Tables S11 and S12).

**Table 3:** Event and non-event NRI for study outcomes for the total cohort. 95% confidence intervals were based on 500 bootstrap samples. (**a**) NRI results for all reclassifications. (**b**) Only reclassifications with clinically meaningful differences in eGFR.

Outcome	Overall NRI^a^, % (95% CI)	Event NRI, % (95% CI)	Non-event NRI, % (95% CI)
(a) All reclassifications occurring across eGFR category thresholds			
KFRT	−9.3 (−10.5 to −8.0)	1.9 (1.0 to 3.3)	−11.2 (−11.3 to −11.2)
All-cause mortality	12.3 (12.1 to 12.5)	22.1 (21.9 to 22.3)	−9.8 (−9.9 to 9.8)
MACE	10.5 (10.3 to 10.7)	20.9 (20.7 to 21.1)	−10.4 (−10.5 to −10.4)
(b) Reclassified with clinically meaningful eGFR difference			
KFRT	−5.5 (−6.2 to −4.7)	2.8 (2.1 to 3.5)	−8.3 (−8.3 to −8.3)
All-cause mortality	9.8 (9.6 to 10.0)	17.0 (16.8 to 17.2)	−7.2 (−7.1 to −7.2)
MACE	7.6 (7.4 to 7.9)	15.4 (15.1 to 15.6)	−7.7 (−7.7 to −7.8)

^a^Note that the denominators for the event and nonevent NRI are different, e.g. the total number of events is much smaller than the total number of nonevents, which complicates interpretation of the overall NRI. Please see Supplemental Methods for further explanation.

**Table 4:** Differences in nephrologist referral, medication eligibility, dose adjustment, and contraindication for common medications using CKD-EPI 2009 or EKFC 2021 for routine eGFR evaluation.

Clinical decision	Population	eGFR threshold (ml/min/1.73 m^2^)	By CKD-EPI *n*	By EKFC *n*	Change, *n* (%)
**(a) All reclassifications occurring across eGFR category thresholds**					
Nephrologist referral	Overall	<30	6 378	7 787	+1 409 (+22.1%)
Medication eligibility or need of dose adjustment					
SGLT2i eligibility	Overall	20–59	77 602	108 323	+30 721 (+39.6%)
Need of apixaban dose reduction	Atrial fibrillation	15–59	9 555	12 194	+2 639 (+27.6%)
Contraindication for treatment					
Spironolactone	Heart failure	<30	2 351	2 961	+610 (+25.9%)
Metformin	Diabetes type 2	<30	1 597	1 845	+248 (+15.5%)
SGLT2is	Overall	<20	1 896	2 059	+163 (+8.6%)
Apixaban	Atrial fibrillation	<15	161	176	+15 (+9.3%)
**(b) Reclassified with clinically meaningful eGFR difference >5 mL/min/1.73 m^2^^a^**					
Medication eligibility or need of dose adjustment					
SGLT2i eligibility	Overall	20–59	77 602	98 427	+20 825 (+26.8%)
Need of apixaban dose reduction	Atrial fibrillation	15–59	9 555	11 480	+1 925 (+20.1%)

^a^There are no results for nephrologist referrals or medication contraindications because no patient with eGFR differences of this magnitude was reclassified around the eGFR thresholds associated with those clinical decisions.

### Clinical implications

If using the EKFC equation, the prevalence of G3–G5 would increase 38% from 4.5% to 6.2%. In addition, 22% more patients would meet criteria for nephrologist referral (Table 4a). The proportion of people with an indication for SGLT2i would increase by 40%, but 9% of users of SGLT2is would become non-eligible.As many as 28% of patients with atrial fibrillation would need to reduce their dose of apixaban and 9% would become non-eligible for anticoagulation. Furthermore, 26% of patients with heart failure would be classified as having a contraindication for spironolactone, and 16% of people with diabetes would meet contraindications for metformin. When limiting the analysis to clinically meaningful reclassifications, the prevalence of G3–G5 would increase 24% from 4.5% to 5.6%. The proportion of participants meeting criteria for SGLT2i use would still increase by 27%, and up to 20% of patients with atrial fibrillation would be recommended for apixaban dose reduction (Table 4b). Because there were no reclassifications with a clinically meaningful eGFR difference in categories G4–G5, there were no differences in nephrologist referral or contraindications to drugs.

## DISCUSSION

We here evaluated the clinical implications of changing from the CKD-EPI to the EKFC eGFR_cr_ equation in a Northern European health system. The generally lower eGFR (median 4.9 ml/min/1.73 m^2^ lower) would lead to 11% of participants being reclassified to a lower eGFR category and <1% reclassified to a higher level. However, lower eGFR at a population level would lead to an increase in the prevalence of CKD G3–G5 from 4.5% to 6.2%, representing a relative increase of 38%, which may affect clinical decisions such as referrals to nephrology or eligibility/contraindication for common medications. Restricting the analysis to meaningful differences in eGFR (>5 ml/min/1.73 m^2^), 8% were reclassified to a lower eGFR, none were reclassified to a higher eGFR, and the prevalence of CKD G3–G5 increased to 5.6%, a relative increase of 24%. Both equations were strong predictors of adverse outcomes. After adjustment for age, participants reclassified to lower eGFR categories had a similar risk of death and cardiovascular disease events than those not reclassified. KFRT results were more heterogenous, and counterintuitively, participants reclassified to lower eGFR categories had a lower risk of KFRT, which was not fully explained by their age.

eGFR reductions were greater in younger (<40 years) and older (≥65 years) participants, and in women (vs. men). Despite their similarities in eGFR values, CKD-EPI and EKFC equations model age and sex differently, as described in detail recently [20]; our observations likely reflect differences in equation structure. Thus, our observations likely reflect differences in equation structure. Our study expands recent observations of Veltkamp *et al.* [10] to a different region and healthcare setting, building on the evidence they found in individuals attending a large hospital in The Netherlands. However, they observed a smaller change in CKD prevalence than in our study (29%), which may be explained by the older age of their participants (median age 51 years vs. 46 years in our study). Additionally, instead of considering only patients accessing outpatient specialist care, our complete health system setting consists mainly of participants who received creatinine testing in connection with a primary care encounter.

The current definition and classification of CKD is based on fixed thresholds of GFR, and thus even small differences in eGFR can still carry important clinical consequences. Our analysis reveals that modest reductions in eGFR would increase the number of individuals meeting nephrology referral criteria and may also affect medication eligibility and dosing for a substantial number of patients. Awareness of these implications is important for European organizations, healthcare professionals, researchers, and policy makers when planning resources related to a potential change from the CKD-EPI to the EKFC equation. However, we note that the differences reported in our modeling may not necessarily have clinical impact: in real-world care, there are many factors beyond eGFR_cr_ that should be considered when making these clinical decisions, such as the underlying cause of CKD, levels of albuminuria, disease severity/symptoms, associated comorbid conditions, co-medications, and expected benefit versus risk of planned treatments. Furthermore, creatinine-based eGFR has known biological and laboratory variability, particularly at higher values, which clinicians should consider when interpreting results [21]. The use of cystatin C for confirmation of eGFR is also increasing; differences between the CKD-EPI and EKFC equations for eGFR_cys_ and eGFR_cr-cys_ are smaller than differences for eGFR_cr_ [20]. Finally, the use of risk prediction instruments that incorporate eGFR plus other variables for outcomes such as KFRT, mortality, and cardiovascular events is increasing, and predicted risk based on multiple variables rather than eGFR category alone will minimize differences between risk attributed to the choice of eGFR equation [22].

Since younger individuals tend to have higher eGFR the large differences we observed between equations in this age group are unlikely to have clinical implications for most of these participants, who remain in category G1 or G2 regardless of equation. Older participants (≥65 years) comprised most of those reclassified to lower eGFR categories. Consequently, reclassified individuals also had more comorbid conditions and used more medications, compared with non-reclassified individuals. Understandably, those who were reclassified had a higher risk of mortality and cardiovascular events than those who were not reclassified after accounting for differences in eGFR, and adjustment for age abrogated these associations. However, these same individuals remained counterintuitively at lower risk for KFRT. Consistent with these findings, we observed that overall NRI for mortality and cardiovascular events was +12.3% and +10.5%, respectively, but was −9.3% for KFRT, as 11.2% of individuals who experienced KFRT were “inappropriately” reclassified to a higher risk KDIGO category by the EKFC equation, and only 1.9% of patients who experienced KFRT were “appropriately” reclassified to a lower risk KDIGO category. However, the overall NRI should be interpreted with caution, since the event and non-event components are calculated from different population subgroups. Since the non-event group is much larger than the event group, the same percentage change in non-event NRI reflects a reclassification of more individuals than an equivalent change in event NRI, which can make the overall NRI appear misleading. Despite our large sample size, the number of KFRT events was still low, especially among reclassified patients. Thus, these findings should be interpreted with caution. Such counterintuitive findings were also reported previously [10], although a lower sample size and lower number of events resulted in broad confidence intervals.

It is unclear why an equation that has shown similar performance or slight improvement (with less bias and greater accuracy against mGFR) from CKD-EPI in select European adult populations, including cohorts from Stockholm [7, 8], would lead to reclassifications that are less reflective of risk for KFRT in our study population. These reclassifications may relate to differences in the mGFR methods used in the equation development populations or how each equation models age, rather than meaningful differences in eGFR accuracy or prognosis. First, urinary clearance of iothalamate, used in the development of CKD-EPI, may exceed plasma clearance of iohexol [23], the method used for EKFC, such that differences in performance may partly reflect alignment with the calibration standard rather than meaningful differences in risk prediction. Second, CKD-EPI and EKFC model age as a continuous variable, but differ in their conceptual approach: while EKFC uses two equations based on the assumption that GFR peaks at age 40 [6], CKD-EPI was derived through statistical fitting to mGFR data without incorporation of a physiological model [4]. Thus, EKFC may align more closely with aging-related outcomes such as death and cardiovascular events due to its physiologic emphasis on age-related GFR decline, but this modeling may be marginally less suited to capturing risk for kidney failure, which may be more strongly influenced by disease-specific progression than by age alone.

Study strengths include a large and region-representative study population with long follow-up, virtually no loss to follow-up, and validated KFRT endpoints. Our study also has limitations. First, we did not have data on race. However, our population is likely predominantly White, as Swedish Government statistics report that 2.5% of the people living in Sweden during the data collection period were born in African countries [24]. Therefore, we apply the White Swedish *Q* values for all individuals. Second, these findings apply to healthcare users of Stockholm and generalization to other regions should be done with caution. Third, our evaluation includes healthcare users that required having their creatinine tested, which may lead to a selected sample, but it is the population where eGFR is estimated to make clinical decisions. Fourth, we required only one creatinine test for inclusion, which differs from the way patients are diagnosed at the clinic, requiring two low consecutive eGFR measurements >90 days apart to establish chronicity. We chose not to apply this criterion to avoid inferring selection bias and maximizing the impact of the clinical decisions modeled [25]. Fifth, KFRT is a rare event; we may still lack statistical power to provide accurate estimates for this outcome. Sixth, measurement variability in eGFR is higher at lower serum creatinine (corresponding to higher eGFR_cr_) for both enzymatic and corrected Jaffe methods [21], and a fixed difference of >5 ml/min/1.73 m² may underrepresent meaningful changes at low eGFR and overrepresent them at high eGFR. However, most reclassifications in our study occurred within the moderate eGFR range, where this threshold likely remains a reasonable proxy for clinical impact. Finally, residual and unknown confounding is inherent to observational research, so causation in the observed associations cannot be established.

## CONCLUSION

In this study, changing from the CKD-EPI to EKFC eGFR_cr_ equation would lower eGFR by a modest amount, increasing the prevalence of moderate/severe CKD and impacting classification along key eGFR thresholds relevant to a variety of clinical decisions. eGFR by both equations strongly predicted outcomes, with individuals reclassified to a lower eGFR category by EKFC having similar risks for mortality and MACE, but a reduced risk for KFRT.

## Supplementary Material

gfaf148_Supplemental_File

## Data Availability

The data used in this article cannot be shared publicly due to the privacy of individuals that participated in the study. The data may be shared upon reasonable request for academic research collaborations fulfilling GDPR, and national and institutional ethics regulations and standards by contacting J.-J.C. (juan.jesus.carrero@ki.se).
