# Supplementary material for: Changing from the CKD-EPI to the EKFC creatinine equation to estimate glomerular filtration rate in adults in a Northern European health system
Source: Nephrol Dial Transplant. 2025 Aug 11;41(2):275–85. doi: 10.1093/ndt/gfaf148 (PMC12957916; doi:10.1093/ndt/gfaf148)

**Supplemental Materials**

[Supplemental methods 2](#_Toc193374757)

[Supplemental Table S1. Definitions of study covariates. 4](#_Toc193374758)

[Supplemental Table S2. Definitions of study outcomes. 4](#_Toc193374759)

[Supplemental Table S3. Baseline characteristics stratified by eGFR category using CKD-EPI 5](#_Toc193374760)

[Supplemental Table S4. Baseline characteristics by eGFR category using EKFC 8](#_Toc193374761)

[Supplemental Table S5. eGFR mean and prevalence by population subgroups 11](#_Toc193374762)

[Supplemental Table S6. eGFR trends of reclassified and non-reclassified participants 12](#_Toc193374763)

[Supplemental Table S7. Characteristics of reclassified CKD G3-G5 13](#_Toc193374764)

[Supplemental Table S8. Age-adjusted characteristics of reclassified and non-reclassified 14](#_Toc193374765)

[Supplemental Table S9. Hazard ratios for reclassified participants 16](#_Toc193374766)

[Supplemental Table S10. Hazard ratios for reclassified participants, with CKD G3-G5 merged. 18](#_Toc193374767)

[Supplemental Table S11. Net reclassification index subgroups 19](#_Toc193374768)

[Supplemental Table S12. Net reclassification index subgroups for meaningful reclassifications 20](#_Toc193374769)

[Supplemental Figure S1. Flow chart of included participants. 21](#_Toc193374770)

[Supplemental Figure S2. Median eGFR across the spectrum of age 22](#_Toc193374771)

[Supplemental Figure S3. eGFR distribution for population subgroups 23](#_Toc193374773)

[Supplemental Figure S4. Hazard ratio splines 26](#_Toc193374777)

[Supplemental Figure S5. Hazard ratio splines adjusted for age, sex, comorbidities, and medications 27](#_Toc193374778)

[Supplemental Figure S6. Hazard ratio splines with revised reference eGFR 28](#_Toc193374778)

[Supplemental Figure S7. Hazard ratio splines with revised reference eGFR adjusted for age sex 29](#_Toc193374778)

[Supplemental Figure S8. Fully adjusted hazard ratio splines with revised reference eGFR 30](#_Toc193374778)

Supplemental methods

*Formulas used to calculate eGFR with the CKD-EPI 2009 and EKFC 2021 equations*

|  | **Age** | **Sex** | **Serum creatinine (μmol/L)** | **Equation** |
| --- | --- | --- | --- | --- |
| CKD-EPI 2009* [1] | ≥18 | Female | SCr ≤ 61.9 | 144 * (SCr/61.9)^-0.329^ * (0.993)^Age^ |
|  |  |  | SCr > 61.9 | 144 * (SCr/61.9)^-1.209^ * (0.993)^Age^ |
|  |  | Male | SCr ≤ 79.6 | 141 * (SCr/79.6)^-0.411^ * (0.993)^Age^ |
|  |  |  | SCr > 79.6 | 141 * (SCr/79.6)^-1.209^ * (0.993)^Age^ |
| EKFC 2021**^†^** [2] | 18-40 | Female | SCr/Q < 1.0 | 107.3 * (SCr/Q)^-0.322^ |
|  |  |  | SCr/Q ≥ 1.0 | 107.3 * (SCr/Q)^-1.132^ |
|  |  | Male | SCr/Q < 1.0 | 107.3 * (SCr/Q)^-0.322^ |
|  |  |  | SCr/Q ≥ 1.0 | 107.3 * (SCr/Q)^-1.132^ |
|  | >40 | Female | SCr/Q < 1.0 | 107.3 * (SCr/Q)^-0.322^ * 0.990^(Age-40)^ |
|  |  |  | SCr/Q ≥ 1.0 | 107.3 * (SCr/Q)^-1.132^ * 0.990^(Age-40)^ |
|  |  | Male | SCr/Q < 1.0 | 107.3 * (SCr/Q)^-0.322^ * 0.990^(Age-40)^ |
|  |  |  | SCr/Q ≥ 1.0 | 107.3 * (SCr/Q)^-1.132^ * 0.990^(Age-40)^ |

*Note that for the CKD-EPI 2009 equation, eGFR was calculated without the race coefficient, corresponding to the ASR-NB equation^3^. Race was not available in our cohort, as Sweden does not allow collecting data on ethnicity to prevent discrimination.

**^†^**Q values correspond to the median SCr values for the age- and sex specific European populations, and can be calculated as follows:

Q values for ages ≤25 years:

Males: Q = exp(3.200 + 0.259 * Age - 0.543 * log(Age) – 0.00763 * Age^2^ + 0.0000790 * Age^3^)

Females: Q = exp(3.080 + 0.177 * Age - 0.223 * log(Age) – 0.00596 * Age^2^ + 0.0000686 * Age^3^)

Q values for ages >25 years:

Males: Q = 80 μmol/L

Females: Q = 62 μmol/L

*Methods for calculation of net reclassification index (NRI)*

We used the formulations proposed by Pencina *et al.* [3] for survival and competing risk data to calculate overall NRI, event NRI, and non-event NRI:


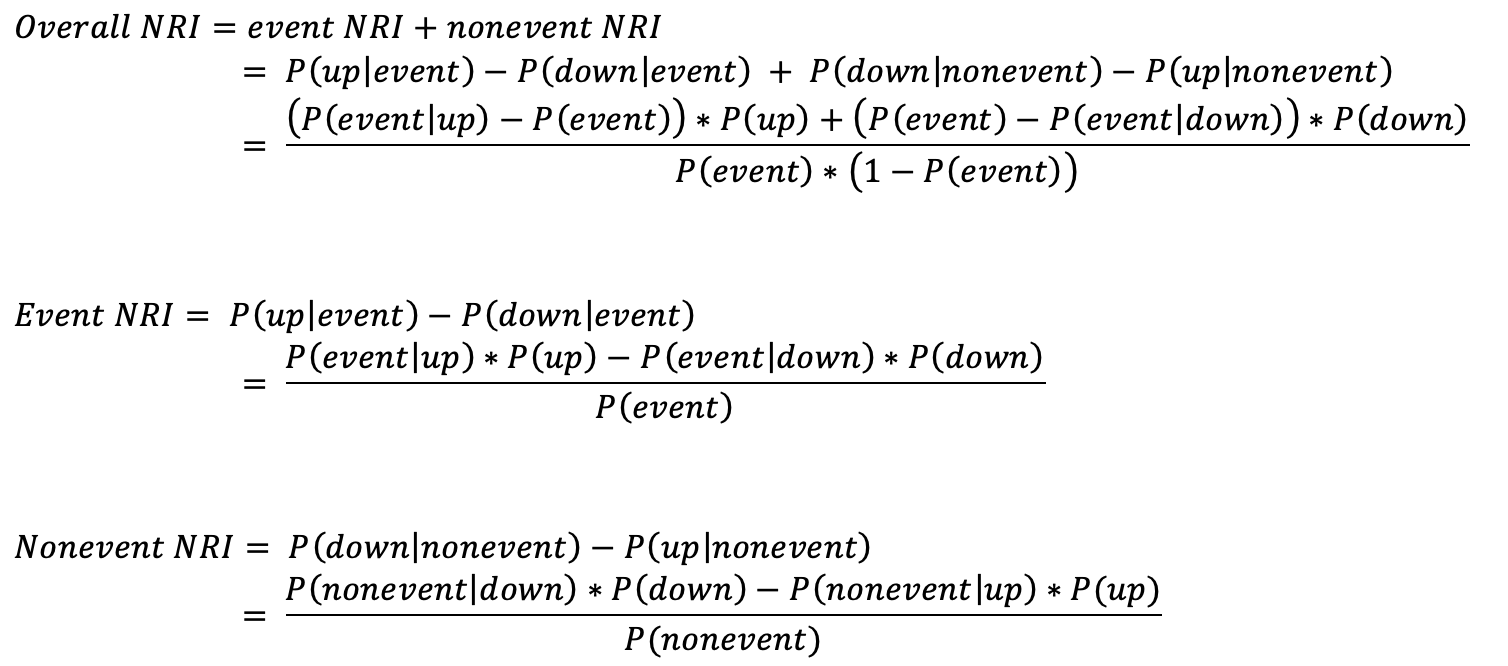


“Up” refers to reclassification to a higher risk, lower absolute range eGFR category and “down” refers to reclassification to a lower risk, higher range eGFR category. The event NRI indicates the net percentage of participants who have the event of interest and are reclassified upward to a higher risk category, and the non-event NRI is the net percentage of individuals without the event of interest who are reclassified downward to a lower risk category. Thus, the event and non-event NRI can range from -100% to +100% [3], reflecting a net percentage of individuals incorrectly (-%) or correctly (+%) reclassified. Overall NRI is calculated as the sum of event and non-event NRI, and it has a theoretical range from -200% to +200. However, overall NRI should not be interpreted as a percentage because the percentages of event and non-event NRI are calculated from two different denominators.

We calculated NRI based on KDIGO GFR categories (e.g. category cutoffs at 90, 60, 45, 30, and 15 mL/min/1.73m^2^), and a risk prediction horizon of 10 years. We used the Kaplan-Meier estimator to handle censored observations for all-cause mortality, and we used the Aalen-Johansen estimator for kidney failure with replacement therapy, and major adverse cardiovascular events to account for the competing risk of non-cardiovascular death. We calculated event, non-event, and overall NRI in the total population, as well as in subgroups of age (<40 year vs. 40 to 64 years vs. ≥65 years), sex, hypertension, diabetes, and cardiovascular disease. We estimated 95% confidence intervals for NRI using 500 bootstrap samples.

*References*

1. Levey AS, Stevens LA, Schmid CH, Zhang YL, Castro AF, 3rd, Feldman HI, et al. A new equation to estimate glomerular filtration rate. Ann Intern Med. 2009;150(9):604-12.
2. Pottel H, Delanaye P. Development and Validation of a Modified Full Age Spectrum Creatinine-Based Equation to Estimate Glomerular Filtration Rate. Ann Intern Med. Jul 2021;174(7):1038. doi:10.7326/l21-0248
3. Pencina MJ, D'Agostino RB, Sr., Steyerberg EW. Extensions of net reclassification improvement calculations to measure usefulness of new biomarkers. Stat Med. 2011;30(1):11-21.

Supplemental Table S1. Definitions of study covariates.

| **Comorbidities^*^** | **ICD-10 codes** | **ATC codes** | |
| --- | --- | --- | --- |
| Myocardial infarction | I200, I21-I22 |  | |
| Other ischemic heart disease | I201, I208, I209, I24, I25 |  | |
| Hypertension | I10-I15 |  | |
| Heart failure | I110, I130, I132, I50 |  | |
| Stroke | I60-I64, I693, I698, I694 |  | |
| Other cerebrovascular disease | I65-I69, G45 (excl G454), G46 |  | |
| Arrhythmia | I44-I49 |  | |
| Peripheral vascular disease | I70, I72, I73 |  | |
| Diabetes mellitus | E10-E14 | A10 | |
| Chronic obstructive pulmonary disease | J44 |  | |
| Cancer in previous year | C00-C43, C45-C97 |  | |
| Liver disease | B18, I850, I859, I982, K70-K77 |  | |
| **Medication^†^** | **ATC codes** |  |  |
| Beta blockers | C07 |  |  |
| Calcium-channel blockers | C08 |  |  |
| Diuretic | C03 |  |  |
| RASi | C09A, C09B, C09C, C09D |  |  |
| Lipid lowering drug | C10 |  |  |
| NSAID | M01A |  |  |

ICD = International Classification of Diseases; ATC = Anatomical Therapeutic Chemical; RASi = renin-angiotensin system inhibition (angiotensin-converting enzyme inhibitor or angiotensin receptor blocker); NSAIDs = non-steroidal anti-inflammatory drugs.

*A comorbidity was considered present if there was an ICD-10 code in general practice, outpatient or inpatient care in any position before the index date.

† Ongoing medications were defined as a dispensation of study drug in the 180 days prior to index date.

Supplemental Table S2. Definitions of study outcomes.

| **Outcomes** | **Definition** |
| --- | --- |
| All-cause mortality | Death in the Swedish Causes of Death Registry |
| Cardiovascular mortality | ICD-10 code of the I family as the main cause of death |
| Myocardial infarction | Hospitalization diagnosis with ICD-10 codes I200, I21, I22 in first or second diagnostic position |
| Stroke | Hospitalization diagnosis with ICD-10 code I63 in first or second diagnostic position |

Supplemental Table S3. Baseline characteristics overall and stratified by eGFR category with the CKD-EPI equation.

|  | **Categories of eGFR with the CKD-EPI equation (mL/min/1.73m^2^)** | | | | | | |
| --- | --- | --- | --- | --- | --- | --- | --- |
| **Characteristic** | **Overall** | **>90**  (G1) | **60-89**  (G2) | **45-59**  (G3a) | **30-44**  (G3b) | **15-29**  (G4) | **<15**  (G5) |
| **Number of individuals** | 1784831 | 1195522 | 509811 | 54017 | 19103 | 5436 | 942 |
| **Mean age (SD), y** | 46.3 (18.6) | 38.5 (13.9) | 59.7 (16.0) | 75.8 (12.6) | 80.5 (11.7) | 79.4 (14.0) | 70.0 (17.8) |
| **Age category, n (%)** |  |  |  |  |  |  |  |
| <20 | 89377 (5.0) | 86525 (7.2) | 2748 (0.5) | 58 (0.1) | 25 (0.1) | 15 (0.3) | 6 (0.6) |
| 20-39 | 663415 (37.2) | 600817 (50.3) | 61349 (12.0) | 860 (1.6) | 203 (1.1) | 118 (2.2) | 68 (7.2) |
| 40-59 | 584073 (32.7) | 400752 (33.5) | 177160 (34.8) | 4700 (8.7) | 916 (4.8) | 371 (6.8) | 174 (18.5) |
| 60-69 | 228894 (12.8) | 93835 (7.8) | 123920 (24.3) | 8817 (16.3) | 1641 (8.6) | 531 (9.8) | 150 (15.9) |
| 70-79 | 128025 (7.2) | 12480 (1.0) | 93827 (18.4) | 16112 (29.8) | 4313 (22.6) | 1088 (20.0) | 205 (21.8) |
| >=80 | 91047 (5.1) | 1113 (0.1) | 50807 (10.0) | 23470 (43.4) | 12005 (62.8) | 3313 (60.9) | 339 (36.0) |
| Female Sex | 947272 (53.1) | 629276 (52.6) | 270287 (53.0) | 32584 (60.3) | 11651 (61.0) | 3083 (56.7) | 391 (41.5) |
| **Mean plasma creatinine (SD), μmol/L†** | 73.1 (20.2) | 66.8 (12.6) | 81.0 (14.0) | 99.8 (16.1) | 127.5 (23.0) | 194.2 (47.4) | 436.8 (159.3) |
| **eGFR with the 2009 equation, mL/min/1.73m^2^** | 97.6 (20.5) | 108.9 (12.3) | 78.9 (7.9) | 53.8 (4.2) | 38.8 (4.2) | 24.4 (4.1) | 10.9 (2.9) |
| **eGFR with the 2021 equation, mL/min/1.73m^2^** | 91.6 (18.5) | 101.9 (9.8) | 74.8 (8.5) | 49.8 (4.5) | 36.1 (4.0) | 23.4 (3.7) | 11.3 (2.8) |
| **Education, n (%)** |  |  |  |  |  |  |  |
| Compulsory school | 293059 (17.0) | 166900 (14.5) | 97366 (19.7) | 18587 (36.3) | 7655 (43.6) | 2230 (45.5) | 321 (36.5) |
| Secondary school | 664839 (38.6) | 447302 (38.8) | 189056 (38.2) | 19643 (38.4) | 6638 (37.8) | 1841 (37.5) | 359 (40.8) |
| University | 764842 (44.4) | 538768 (46.7) | 208816 (42.2) | 12970 (25.3) | 3253 (18.5) | 835 (17.0) | 200 (22.7) |
| **Medical history, n (%)** | 0.0 (0.0) | 0.0 (0.0) | 0.0 (0.0) | 0.0 (0.0) | 0.0 (0.0) | 0.0 (0.0) | 0.0 (0.0) |
| Hypertension | 217406 (12.2) | 71830 (6.0) | 107650 (21.1) | 23853 (44.2) | 10398 (54.4) | 3107 (57.2) | 568 (60.3) |
| Myocardial infarction | 28991 (1.6) | 6907 (0.6) | 14058 (2.8) | 4403 (8.2) | 2551 (13.4) | 948 (17.4) | 124 (13.2) |
| Other ischemic heart disease | 61339 (3.4) | 13613 (1.1) | 31397 (6.2) | 9595 (17.8) | 4884 (25.6) | 1655 (30.4) | 195 (20.7) |
| Heart failure | 37372 (2.1) | 4716 (0.4) | 16486 (3.2) | 8235 (15.2) | 5584 (29.2) | 2116 (38.9) | 235 (24.9) |
| Stroke | 33815 (1.9) | 8189 (0.7) | 16669 (3.3) | 5202 (9.6) | 2742 (14.4) | 896 (16.5) | 117 (12.4) |
| Other cerebrovascular disease | 30227 (1.7) | 7274 (0.6) | 14984 (2.9) | 4711 (8.7) | 2397 (12.5) | 766 (14.1) | 95 (10.1) |
| Arrhythmia | 72682 (4.1) | 21242 (1.8) | 34392 (6.7) | 10162 (18.8) | 5124 (26.8) | 1601 (29.5) | 161 (17.1) |
| Peripheral vascular disease | 16427 (0.9) | 4570 (0.4) | 7330 (1.4) | 2423 (4.5) | 1492 (7.8) | 533 (9.8) | 79 (8.4) |
| Diabetes mellitus | 86036 (4.8) | 40038 (3.3) | 32728 (6.4) | 7784 (14.4) | 3889 (20.4) | 1362 (25.1) | 235 (24.9) |
| Cancer in previous year | 40588 (2.3) | 15552 (1.3) | 19338 (3.8) | 3593 (6.7) | 1557 (8.2) | 466 (8.6) | 82 (8.7) |
| Chronic obstructive pulmonary disease | 26143 (1.5) | 8238 (0.7) | 12813 (2.5) | 3060 (5.7) | 1487 (7.8) | 497 (9.1) | 48 (5.1) |
| Liver disease | 20802 (1.2) | 14266 (1.2) | 5300 (1.0) | 751 (1.4) | 337 (1.8) | 109 (2.0) | 39 (4.1) |
| **Concomitant medications, n (%)** |  |  |  |  |  |  |  |
| Beta blocker | 167043 (9.4) | 53526 (4.5) | 81806 (16.0) | 19620 (36.3) | 8896 (46.6) | 2724 (50.1) | 471 (50.0) |
| Calcium channel blocker | 81630 (4.6) | 25168 (2.1) | 41481 (8.1) | 9270 (17.2) | 3960 (20.7) | 1363 (25.1) | 388 (41.2) |
| Diuretic | 112881 (6.3) | 23630 (2.0) | 54569 (10.7) | 19526 (36.1) | 10946 (57.3) | 3630 (66.8) | 580 (61.6) |
| ACEi/ARB | 154134 (8.6) | 50947 (4.3) | 75063 (14.7) | 17111 (31.7) | 7990 (41.8) | 2594 (47.7) | 429 (45.5) |
| Lipid lowering drug | 116673 (6.5) | 37229 (3.1) | 60759 (11.9) | 12153 (22.5) | 4816 (25.2) | 1470 (27.0) | 246 (26.1) |
| NSAID | 219525 (12.3) | 137413 (11.5) | 70827 (13.9) | 7944 (14.7) | 2620 (13.7) | 656 (12.1) | 65 (6.9) |
| Calendar Year |  |  |  |  |  |  |  |
| 2007-2010 | 1046559 (58.6) | 629511 (52.7) | 348369 (68.3) | 45871 (84.9) | 17188 (90.0) | 4855 (89.3) | 765 (81.2) |
| 2011-2014 | 321464 (18.0) | 246533 (20.6) | 70077 (13.7) | 3625 (6.7) | 901 (4.7) | 256 (4.7) | 72 (7.6) |
| 2015-2019 | 248477 (13.9) | 186020 (15.6) | 58692 (11.5) | 2914 (5.4) | 607 (3.2) | 180 (3.3) | 64 (6.8) |
| 2020-2021 | 168331 (9.4) | 133458 (11.2) | 32673 (6.4) | 1607 (3.0) | 407 (2.1) | 145 (2.7) | 41 (4.4) |

y = years; n = number; eGFR = estimated glomerular filtration rate; RASi = renin-angiotensin system inhibition (angiotensin-converting enzyme inhibitor or angiotensin receptor blocker); NSAIDs = non-steroidal anti-inflammatory drugs.

† Mean (SD) plasma creatinine in mg/dL is 0.83 (0.23) for overall, 0.75 (0.14) for eGFR ≥90, 0.92 (0.16) for eGFR 60-89, 1.13 (0.18) for eGFR 45-59, 1.44 (0.26) for eGFR 30-44, 2.19 (0.54) for eGFR 15-29 and 4.94 (1.80) for eGFR <15. To convert plasma creatinine from μmol/L to mg/dL, multiply by 0.0113.

Supplemental Table S4. Baseline characteristics overall and stratified by eGFR category with the EKFC creatinine-based eGFR equation.

|  | **Categories of eGFR with the EKFC equation (mL/min/1.73m^2^)** | | | | | | |
| --- | --- | --- | --- | --- | --- | --- | --- |
| **Characteristic** | **Overall** | **>90**  (G1) | **60-89**  (G2) | **45-59**  (G3a) | **30-44**  (G3b) | **15-29**  (G4) | **<15**  (G5) |
| **Number of individuals** | 1784831 | 1037041 | 637408 | 75665 | 26930 | 6874 | 913 |
| **Mean age (SD), y** | 46.3 (18.6) | 36.7 (11.9) | 56.5 (17.0) | 75.7 (11.9) | 81.3 (10.8) | 81.1 (13.2) | 72.1 (17.6) |
| **Age category, n (%)** |  |  |  |  |  |  |  |
| <20 | 89377 (5.0) | 70890 (6.8) | 18242 (2.9) | 172 (0.2) | 48 (0.2) | 17 (0.2) | 8 (0.9) |
| 20-39 | 663415 (37.2) | 564892 (54.5) | 97087 (15.2) | 1045 (1.4) | 214 (0.8) | 124 (1.8) | 53 (5.8) |
| 40-59 | 584073 (32.7) | 373802 (36.0) | 203818 (32.0) | 5063 (6.7) | 904 (3.4) | 350 (5.1) | 136 (14.9) |
| 60-69 | 228894 (12.8) | 26725 (2.6) | 186268 (29.2) | 13246 (17.5) | 1975 (7.3) | 546 (7.9) | 134 (14.7) |
| 70-79 | 128025 (7.2) | 707 (0.1) | 94430 (14.8) | 25279 (33.4) | 6152 (22.8) | 1252 (18.2) | 205 (22.5) |
| >=80 | 91047 (5.1) | 25 (0.0) | 37563 (5.9) | 30860 (40.8) | 17637 (65.5) | 4585 (66.7) | 377 (41.3) |
| Female Sex | 947272 (53.1) | 541715 (52.2) | 336853 (52.8) | 47004 (62.1) | 17186 (63.8) | 4113 (59.8) | 401 (43.9) |
| **Mean plasma creatinine (SD), μmol/L†** | 73.1 (20.2) | 66.3 (12.4) | 78.3 (14.6) | 93.1 (16.6) | 118.2 (23.8) | 184.0 (51.4) | 434.0 (165.1) |
| **eGFR with the 2009 equation, mL/min/1.73m^2^** | 97.6 (20.5) | 111.1 (11.5) | 83.5 (8.9) | 58.4 (5.0) | 42.2 (5.0) | 25.8 (4.7) | 10.8 (2.9) |
| **eGFR with the 2021 equation, mL/min/1.73m^2^** | 91.6 (18.5) | 104.2 (8.3) | 78.5 (8.0) | 53.7 (4.2) | 38.9 (4.2) | 24.5 (4.0) | 11.1 (2.7) |
| **Education, n (%)** |  |  |  |  |  |  |  |
| Compulsory school | 293059 (17.0) | 137611 (13.8) | 115922 (18.7) | 25302 (35.2) | 11040 (44.4) | 2861 (46.6) | 323 (38.1) |
| Secondary school | 664839 (38.6) | 385617 (38.6) | 239545 (38.7) | 27759 (38.6) | 9303 (37.4) | 2265 (36.9) | 350 (41.3) |
| University | 764842 (44.4) | 476100 (47.6) | 264141 (42.6) | 18906 (26.3) | 4503 (18.1) | 1017 (16.6) | 175 (20.6) |
| **Medical history, n (%)** |  |  |  |  |  |  |  |
| Hypertension | 217406 (12.2) | 47104 (4.5) | 120184 (18.9) | 31449 (41.6) | 14184 (52.7) | 3926 (57.1) | 559 (61.2) |
| Myocardial infarction | 28991 (1.6) | 4145 (0.4) | 14832 (2.3) | 5380 (7.1) | 3305 (12.3) | 1197 (17.4) | 132 (14.5) |
| Other ischemic heart disease | 61339 (3.4) | 7599 (0.7) | 32713 (5.1) | 12088 (16.0) | 6633 (24.6) | 2099 (30.5) | 207 (22.7) |
| Heart failure | 37372 (2.1) | 2586 (0.2) | 15210 (2.4) | 9391 (12.4) | 7216 (26.8) | 2716 (39.5) | 253 (27.7) |
| Stroke | 33815 (1.9) | 4998 (0.5) | 17239 (2.7) | 6597 (8.7) | 3685 (13.7) | 1178 (17.1) | 118 (12.9) |
| Other cerebrovascular disease | 30227 (1.7) | 4435 (0.4) | 15498 (2.4) | 5941 (7.9) | 3275 (12.2) | 982 (14.3) | 96 (10.5) |
| Arrhythmia | 72682 (4.1) | 15239 (1.5) | 35231 (5.5) | 12936 (17.1) | 7026 (26.1) | 2074 (30.2) | 176 (19.3) |
| Peripheral vascular disease | 16427 (0.9) | 3033 (0.3) | 7730 (1.2) | 3035 (4.0) | 1870 (6.9) | 678 (9.9) | 81 (8.9) |
| Diabetes mellitus | 86036 (4.8) | 30521 (2.9) | 38722 (6.1) | 9849 (13.0) | 5099 (18.9) | 1610 (23.4) | 235 (25.7) |
| Cancer in previous year | 40588 (2.3) | 10512 (1.0) | 22476 (3.5) | 4844 (6.4) | 2100 (7.8) | 578 (8.4) | 78 (8.5) |
| Chronic obstructive pulmonary disease | 26143 (1.5) | 4621 (0.4) | 14765 (2.3) | 4084 (5.4) | 1999 (7.4) | 625 (9.1) | 49 (5.4) |
| Liver disease | 20802 (1.2) | 12462 (1.2) | 6826 (1.1) | 940 (1.2) | 409 (1.5) | 130 (1.9) | 35 (3.8) |
| **Concomitant medications, n (%)** |  |  |  |  |  |  |  |
| Beta blocker | 167043 (9.4) | 35800 (3.5) | 89772 (14.1) | 25585 (33.8) | 12004 (44.6) | 3430 (49.9) | 452 (49.5) |
| Calcium channel blocker | 81630 (4.6) | 15408 (1.5) | 46409 (7.3) | 12381 (16.4) | 5410 (20.1) | 1655 (24.1) | 367 (40.2) |
| Diuretic | 112881 (6.3) | 14168 (1.4) | 55283 (8.7) | 23664 (31.3) | 14543 (54.0) | 4646 (67.6) | 577 (63.2) |
| ACEi/ARB | 154134 (8.6) | 33324 (3.2) | 84891 (13.3) | 21844 (28.9) | 10486 (38.9) | 3184 (46.3) | 405 (44.4) |
| Lipid lowering drug | 116673 (6.5) | 22370 (2.2) | 69281 (10.9) | 16507 (21.8) | 6533 (24.3) | 1746 (25.4) | 236 (25.8) |
| NSAID | 219525 (12.3) | 116131 (11.2) | 87634 (13.7) | 11181 (14.8) | 3693 (13.7) | 818 (11.9) | 68 (7.4) |
| Calendar Year |  |  |  |  |  |  |  |
| 2007-2010 | 1046559 (58.6) | 532496 (51.3) | 418910 (65.7) | 63929 (84.5) | 24269 (90.1) | 6201 (90.2) | 754 (82.6) |
| 2011-2014 | 321464 (18.0) | 220500 (21.3) | 93998 (14.7) | 5330 (7.0) | 1273 (4.7) | 297 (4.3) | 66 (7.2) |
| 2015-2019 | 248477 (13.9) | 164158 (15.8) | 79116 (12.4) | 4089 (5.4) | 848 (3.1) | 209 (3.0) | 57 (6.2) |
| 2020-2021 | 168331 (9.4) | 119887 (11.6) | 45384 (7.1) | 2317 (3.1) | 540 (2.0) | 167 (2.4) | 36 (3.9) |

Supplemental Table S5. Mean estimated GFR and proportion of individuals with eGFR <60, <45 and <30 mL/min/1.73m^2^ overall and in subgroups with the CKD-EPI and EKFC equations.

|  | **eGFR with the CKD-EPI equation** | | | | **eGFR with the EKFC equation** | | | |
| --- | --- | --- | --- | --- | --- | --- | --- | --- |
|  | **Mean value, mL/min/1.73m^2^** | **% with eGFR <60 mL/min/ 1.73m^2^** | **% with eGFR <45 mL/min/ 1.73m^2^** | **% with eGFR <30 mL/min/ 1.73m^2^** | **Mean value, mL/min/1.73m^2^** | **% with eGFR <60 mL/min/ 1.73m^2^** | **% with eGFR <45 mL/min/ 1.73m^2^** | **% with eGFR <30 mL/min/ 1.73m^2^** |
| **Overall** | 98 | 4.5 | 1.4 | 0.4 | 92 | 6.2 | 1.9 | 0.4 |
| **Age ≥ 65 years** | 73 | 21.3 | 7.1 | 1.7 | 66 | 30.3 | 10.0 | 2.1 |
| **Age < 65 years** | 103 | 0.8 | 0.2 | 0.1 | 97 | 1.0 | 0.2 | 0.1 |
| **Male** | 97 | 3.8 | 1.2 | 0.3 | 92 | 5.0 | 1.6 | 0.4 |
| **Female** | 98 | 5.0 | 1.6 | 0.4 | 91 | 7.3 | 2.3 | 0.5 |
| **Hypertension yes** | 79 | 17.4 | 6.5 | 1.7 | 74 | 23.1 | 8.6 | 2.1 |
| **Hypertension no** | 100 | 2.7 | 0.7 | 0.2 | 94 | 3.8 | 1.0 | 0.2 |
| **Diabetes yes** | 86 | 15.4 | 6.4 | 1.9 | 80 | 19.5 | 8.1 | 2.1 |
| **Diabetes no** | 98 | 3.9 | 1.2 | 0.3 | 92 | 5.5 | 1.6 | 0.3 |
| **Cardiovascular disease* yes** | 77 | 22.2 | 8.9 | 2.3 | 71 | 29.1 | 11.9 | 2.9 |
| **Cardiovascular disease* no** | 100 | 2.6 | 0.7 | 0.2 | 94 | 3.8 | 0.9 | 0.2 |

eGFR = estimated glomerular filtration rate. Cardiovascular disease was defined as a composite of myocardial infarction, ischemic heart disease, heart failure, stroke, other cerebrovascular disease, arrhythmia and peripheral vascular disease.

Supplemental Table S6. Mean eGFR and eGFR difference between reclassified and non-reclassified individuals when changing from the CKD-EPI to EKFC equation, stratified by eGFR category.

| **eGFR category^*^**  (G category) | **eGFR equation** | **Reclassified upward, mean eGFR^*^** | **Mean eGFR difference^*^** | **Not reclassified, mean eGFR^*^** | **Reclassified downward, mean eGFR^*^** | **Mean eGFR difference^*^** |
| --- | --- | --- | --- | --- | --- | --- |
| **>90** (G1) | CKD-EPI | - | - | 111 | 94 | -17 |
|  | EKFC | - | - | 104 | 87 | -17 |
| **60-89** (G2) | CKD-EPI | 89 | +19 | 80 | 63 | -17 |
|  | EKFC | 91 | +25 | 76 | 57 | -19 |
| **45-49** (G3a) | CKD-EPI | 59 | +4 | 55 | 47 | -8 |
|  | EKFC | 61 | +10 | 51 | 43 | -8 |
| **30-44** (G3b) | CKD-EPI | 44 | +5 | 39 | 31 | -8 |
|  | EKFC | 46 | +9 | 37 | 29 | -8 |
| **15-29** (G4) | CKD-EPI | 29 | +5 | 24 | 15 | -9 |
|  | EKFC | 31 | +8 | 23 | 15 | -8 |
| **<15** (G5) | CKD-EPI | 14 | +3 | 11 | - | - |
|  | EKFC | 16 | +5 | 11 | - | - |

eGFR = estimated glomerular filtration rate.

* in mL/min/1.73m^2^

Supplemental Table S7. Baseline characteristics of reclassified and non-reclassified individuals when changing from the CKD-EPI to EKFC equation for individuals classified to CKD G3-5 with CKD-EPI 2009. (*n*=number; eGFR=estimated glomerular filtration rate; RASi=renin-angiotensin system inhibition (angiotensin-converting enzyme inhibitor or angiotensin receptor blocker); NSAIDs=non-steroidal anti-inflammatory drugs.

|  | Individuals in G3-5 with the 2009 CKD-EPI equation (*N*=79,498) | | |
| --- | --- | --- | --- |
| **Characteristic** | Reclassified to higher eGFR category  (n = 615) | Not reclassified  (n = 68016) | Reclassified to lower eGFR category  (n = 10867) |
| **Mean age (SD), y** | 46 (7.2) | 76.2 (12.7) | 84.3 (8.4) |
| **Age category, (%)** |  |  |  |
| <20 | 0 (0.0) | 71 (0.1) | 33 (0.3) |
| 20-39 | 108 (17.6) | 1120 (1.6) | 21 (0.2) |
| 40-59 | 476 (77.4) | 5676 (8.3) | 9 (0.1) |
| 60-69 | 29 (4.7) | 10765 (15.8) | 345 (3.2) |
| 70-79 | 2 (0.3) | 19547 (28.7) | 2169 (20.0) |
| ≥80 | 0 (0.0) | 30837 (45.3) | 8290 (76.3) |
| **Female sex, n (%)** | 154 (25.0) | 39858 (58.6) | 7697 (70.8) |
| **Mean plasma creatinine (SD), μmol/L†** | 164.4 (80.0) | 117.9 (53.6) | 108.2 (22.2) |
| **eGFR with the CKD-EPI equation, mL/min/1.73m^2^** | 47.7 (16.0) | 48.1 (11.2) | 45.2 (6.0) |
| **eGFR with the EKFC equation, mL/min/1.73m^2^** | 49.3 (16.1) | 44.7 (10.3) | 41.0 (5.3) |
| **Education, n (%)** |  |  |  |
| Compulsory school | 83 (14.0) | 24011 (37.5) | 4699 (47.4) |
| Secondary school | 223 (37.7) | 24682 (38.6) | 3576 (36.0) |
| University | 285 (48.2) | 15328 (23.9) | 1645 (16.6) |
| **Medical history, n (%)** |  |  |  |
| Hypertension | 162 (26.3) | 32254 (47.4) | 5510 (50.7) |
| Myocardial infarction | 11 (1.8) | 6732 (9.9) | 1283 (11.8) |
| Other ischemic heart disease | 15 (2.4) | 13628 (20.0) | 2686 (24.7) |
| Heart failure | 26 (4.2) | 13241 (19.5) | 2903 (26.7) |
| Stroke | 21 (3.4) | 7412 (10.9) | 1524 (14.0) |
| Other cerebrovascular disease | 14 (2.3) | 6633 (9.8) | 1322 (12.2) |
| Arrhythmia | 19 (3.1) | 14124 (20.8) | 2905 (26.7) |
| Peripheral vascular disease | 10 (1.6) | 3837 (5.6) | 680 (6.3) |
| Diabetes | 64 (10.4) | 11452 (16.8) | 1754 (16.1) |
| Cancer | 25 (4.1) | 4899 (7.2) | 774 (7.1) |
| Chronic obstructive pulmonary disease | 9 (1.5) | 4304 (6.3) | 779 (7.2) |
| Liver disease | 24 (3.9) | 1096 (1.6) | 116 (1.1) |
| **Concomitant medications, n (%)** |  |  |  |
| Beta blocker | 122 (19.8) | 27036 (39.7) | 4553 (41.9) |
| Calcium channel blocker | 99 (16.1) | 12838 (18.9) | 2044 (18.8) |
| Diuretic | 116 (18.9) | 28854 (42.4) | 5712 (52.6) |
| RASi | 160 (26.0) | 24247 (35.6) | 3717 (34.2) |
| Lipid lowering drug | 59 (9.6) | 16342 (24.0) | 2284 (21.0) |
| NSAIDs | 109 (17.7) | 9735 (14.3) | 1441 (13.3) |
| **Calendar year, n (%)** |  |  |  |
| 2007-2010 | 347 (56.4) | 58416 (85.9) | 9916 (91.2) |
| 2011-2014 | 108 (17.6) | 4279 (6.3) | 467 (4.3) |
| 2015-2019 | 104 (16.9) | 3355 (4.9) | 306 (2.8) |
| 2020-2021 | 56 (9.1) | 1966 (2.9) | 178 (1.6) |

Supplemental Table S8. Baseline characteristics of reclassified and non-reclassified individuals when changing from the CKD-EPI to EKFC equation after adjusting for age with inverse probability weighting.

| **Characteristic** | **Reclassified to higher eGFR category**  **(n = 3777)** | **Reclassified to lower eGFR category**  **(n = 203758)** | **Not reclassified**  **(n = 1577296)** |
| --- | --- | --- | --- |
| **Weighted population, N** | 1716574.9 | 68044.5 | 1716574.9 |
| **Mean age (SD), y** | 46.3 (20.6) | 41.9 (3.4) | 46.3 (18.4) |
| **Age category, n (%)** | 89408.8 (5.2) | 0.0 (0.0) | 89378.5 (5.0) |
| <50 | 663024.6 (38.6) | 16774.3 (24.7) | 663475.5 (37.2) |
| 50-59 | 516294.6 (30.1) | 51167.7 (75.2) | 584013.9 (32.7) |
| 60-69 | 228899.7 (13.3) | 92.3 (0.1) | 228916.4 (12.8) |
| 70-79 | 128176.6 (7.5) | 10.3 (0.0) | 128034.5 (7.2) |
| ≥80 | 90770.6 (5.3) | 0.0 (0.0) | 91011.9 (5.1) |
| **Female sex, n (%)** | 1001201.3 (58.3) | 6408.6 (9.4) | 941490.1 (52.7) |
| **Mean plasma creatinine (SD), μmol/L†** | 77.4 (14.6) | 100.9 (39.1) | 73.2 (21.0) |
| **eGFR with the CKD-EPI equation, mL/min/1.73m^2^** | 90.2 (11.8) | 83.1 (15.8) | 97.9 (21.2) |
| **eGFR with the EKFC equation, mL/min/1.73m^2^** | 83.4 (11.0) | 84.4 (15.7) | 91.9 (18.9) |
| **Education, n (%)** |  |  |  |
| Compulsory school | 274117.2 (16.5) | 6193.6 (9.4) | 293137.8 (17.0) |
| Secondary school | 644056.8 (38.7) | 25202.2 (38.1) | 663943.0 (38.5) |
| University | 744129.1 (44.8) | 34790.0 (52.6) | 765595.4 (44.4) |
| **Medical history, n (%)** |  |  |  |
| Hypertension | 226392.8 (13.2) | 4835.7 (7.1) | 217849.5 (12.2) |
| Myocardial infarction | 29748.4 (1.7) | 373.0 (0.5) | 29293.3 (1.6) |
| Other ischemic heart disease | 64801.5 (3.8) | 396.6 (0.6) | 61661.1 (3.5) |
| Heart failure | 36998.1 (2.2) | 610.4 (0.9) | 37684.4 (2.1) |
| Stroke | 34238.9 (2.0) | 600.4 (0.9) | 33807.0 (1.9) |
| Other cerebrovascular disease | 30473.6 (1.8) | 415.0 (0.6) | 30355.5 (1.7) |
| Arrhythmia | 72468.9 (4.2) | 1522.5 (2.2) | 73094.9 (4.1) |
| Peripheral vascular disease | 16810.9 (1.0) | 297.7 (0.4) | 16510.4 (0.9) |
| Diabetes | 79576.7 (4.6) | 1717.9 (2.5) | 86647.9 (4.9) |
| Cancer | 41449.0 (2.4) | 825.3 (1.2) | 40702.6 (2.3) |
| Chronic obstructive pulmonary disease | 28887.8 (1.7) | 174.9 (0.3) | 25693.7 (1.4) |
| Liver disease | 15457.8 (0.9) | 896.7 (1.3) | 21071.6 (1.2) |
| **Concomitant medications, n (%)** |  |  |  |
| Beta blocker | 171528.0 (10.0) | 3561.3 (5.2) | 168302.2 (9.4) |
| Calcium channel blocker | 85778.2 (5.0) | 2300.8 (3.4) | 81746.8 (4.6) |
| Diuretic | 116847.2 (6.8) | 1908.5 (2.8) | 113671.8 (6.4) |
| RASi | 158426.0 (9.2) | 4269.7 (6.3) | 155357.7 (8.7) |
| Lipid lowering drug | 122167.7 (7.1) | 2002.8 (2.9) | 117498.4 (6.6) |
| NSAIDs | 207675.8 (12.1) | 8489.8 (12.5) | 219140.0 (12.3) |
| **Calendar year, n (%)** |  |  |  |
| 2007-2010 | 963645.9 (56.1) | 34690.0 (51.0) | 1049201.3 (58.8) |
| 2011-2014 | 302082.8 (17.6) | 13738.1 (20.2) | 321951.0 (18.0) |
| 2015-2019 | 281869.6 (16.4) | 12452.9 (18.3) | 245744.1 (13.8) |
| 2020-2021 | 168976.6 (9.8) | 7163.4 (10.5) | 167934.4 (9.4) |

y = years; n = number; eGFR = estimated glomerular filtration rate; RASi = renin-angiotensin system inhibition (angiotensin-converting enzyme inhibitor or angiotensin receptor blocker); NSAIDs = non-steroidal anti-inflammatory drugs.

† Mean (SD) plasma creatinine is 0.90 (0.24) for reclassified and 0.83 (0.26) for non-reclassified individuals. To convert plasma creatinine from μmol/L to mg/dL, multiply by 0.0113.

Supplemental Table S9. Hazard ratios comparing reclassified and non-reclassified participants across eGFR categories using the CKD-EPI equation. Results are shown for three models: unadjusted, adjusted for CKD-EPI eGFR, and adjusted for CKD-EPI eGFR and age. Comparisons include participants reclassified to a lower eGFR category versus those not reclassified, those reclassified downward with a clinically meaningful eGFR difference versus both non-reclassified participants and those reclassified with a small difference, and participants reclassified to a higher eGFR category versus non-reclassified individuals. Cell colors indicate the direction of risk: grey for no difference, orange for increased risk among reclassified participants, and green for reduced risk.

| **eGFR category,**  mL/min/1.73m^2^ (G category)  (eGFR in mL/min/1.73m^2^) | | **Model covariates** | **↑ eGFR category** | **↓ eGFR category** | **↓ eGFR category**  (with meaningful difference) |
| --- | --- | --- | --- | --- | --- |
| **KFRT,** HR (95% CI) | | | | | |
| **>90** (G1) | Unadjusted | | **-** | 1.74 (1.41-2.16) | 1.71 (1.35-2.18) |
|  | eGFR | | **-** | 1.38 (1.07-1.79) | 1.35 (1.04-1.76) |
|  | eGFR, age | | **-** | 1.13 (0.87-1.48) | 1.02 (0.77-1.35) |
|  | Number of events (n) | | **-** | 108 | 81 |
| **60-89** (G2) | Unadjusted | | 0.76 (0.38-1.52) | 1.88 (1.42-2.50) | 0.58 (0.3-1.11) |
|  | eGFR | | 1.29 (0.64-2.59) | 0.49 (0.36-0.68) | 0.18 (0.09-0.35) |
|  | eGFR, age | | 1.09 (0.54-2.20) | 0.70 (0.51-0.97) | 0.30 (0.15-0.58) |
|  | Number of events (n) | | 2 | 53 | 9 |
| **45-59** (G3a) | Unadjusted | | 1.42 (0.98-2.07) | 0.42 (0.27-0.66) | 0.50 (0.19-1.35) |
|  | eGFR | | 2.59 (1.76-3.82) | 0.10 (0.06-0.16) | 0.28 (0.11-0.77) |
|  | eGFR, age | | 1.19 (0.80-1.75) | 0.23 (0.14-0.37) | 0.33 (0.12-0.90) |
|  | Number of events (n) | | 7 | 20 | 4 |
| **30-44** (G3b) | Unadjusted | | 1.56 (1.19-2.03) | 0.69 (0.41-1.15) | **-** |
|  | eGFR | | 2.35 (1.78-3.09) | 0.23 (0.13-0.39) | **-** |
|  | eGFR, age | | 0.95 (0.72-1.25) | 0.58 (0.34-0.99) | **-** |
|  | Number of events (n) | | 14 | 15 |  |
| **15-29** (G4) | Unadjusted | | 1.33 (1.10-1.59) | 1.63 (0.52-5.06) | **-** |
|  | eGFR | | 2.21 (1.81-2.69) | 0.40 (0.13-1.24) | **-** |
|  | eGFR, age | | 1.23 (1.01-1.50) | 0.25 (0.08-0.78) | **-** |
|  | Number of events (n) | | 30 | 3 |  |
| **<15** (G5) | Unadjusted | | 1.01 (0.86-1.18) | **-** | **-** |
|  | eGFR | | 1.43 (1.20-1.72) | **-** | **-** |
|  | eGFR, age | | 1.08 (0.90-1.31) | **-** | **-** |
|  | Number of events (n) | | 43 | **-** | **-** |
| All-cause mortality, HR (95% CI) | | | | | |
| **>90** (G1) | Unadjusted | | **-** | 4.48 (4.40-4.56) | 5.17 (5.08-5.26) |
|  | eGFR | | **-** | 2.47 (2.42-2.53) | 2.97 (2.91-3.03) |
|  | eGFR, age | | **-** | 1.04 (1.02-1.07) | 0.99 (0.96-1.01) |
|  | Number of events (n) | | **-** | 23006 | 20546 |
| **60-89** (G2) | Unadjusted | | 0.25 (0.22-0.29) | 3.73 (3.67-3.78) | 5.42 (5.33-5.51) |
|  | eGFR | | 0.28 (0.24-0.33) | 2.67 (2.62-2.73) | 3.82 (3.74-3.90) |
|  | eGFR, age | | 1.16 (0.99-1.35) | 1.02 (1.00-1.04) | 0.96 (0.94-0.98) |
|  | Number of events (n) | | 41 | 20078 | 16450 |
| **45-59** (G3a) | Unadjusted | | 0.26 (0.21-0.34) | 2.11 (2.06-2.17) | 3.35 (3.23-3.48) |
|  | eGFR | | 0.27 (0.21-0.34) | 2.05 (1.97-2.12) | 2.76 (2.65-2.88) |
|  | eGFR, age | | 1.22 (0.96-1.56) | 0.91 (0.88-0.95) | 0.92 (0.88-0.96) |
|  | Number of events (n) | | 17 | 8105 | 2950 |
| **30-44** (G3b) | Unadjusted | | 0.34 (0.27-0.43) | 1.84 (1.74-1.94) | **-** |
|  | eGFR | | 0.35 (0.28-0.44) | 1.66 (1.55-1.77) | **-** |
|  | eGFR, age | | 1.15 (0.91-1.47) | 0.95 (0.89-1.02) | **-** |
|  | Number of events (n) | | 17 | 1430 | **-** |
| **15-29** (G4) | Unadjusted | | 0.47 (0.39-0.56) | 1.95 (1.43-2.66) | **-** |
|  | eGFR | | 0.46 (0.39-0.55) | 2.00 (1.45-2.75) | **-** |
|  | eGFR, age | | 1.06 (0.88-1.28) | 1.17 (0.85-1.60) | **-** |
|  | Number of events (n) | | 30 | 40 | **-** |
| **<15** (G5) | Unadjusted | | 0.56 (0.46-0.68) | **-** | **-** |
|  | eGFR | | 0.48 (0.39-0.59) | **-** | **-** |
|  | eGFR, age | | 0.98 (0.79-1.21) | **-** | **-** |
|  | Number of events (n) | | 27 | **-** | **-** |
| MACE, HR (95% CI) | | | | | |
| **>90** (G1) | Unadjusted | | - | 3.84 (3.77-3.93) | 4.09 (4.00-4.18) |
|  | eGFR | | - | 1.63 (1.59-1.67) | 1.9 (1.86-1.95) |
|  | eGFR, age | | - | 0.74 (0.72-0.77) | 0.68 (0.66-0.70) |
|  | Number of events (n) | | - | 14133 | 11851 |
| **60-89** (G2) | Unadjusted | | 0.40 (0.35-0.45) | 3.40 (3.34-3.47) | 4.69 (4.59-4.80) |
|  | eGFR | | 0.45 (0.40-0.50) | 2.36 (2.30-2.43) | 3.20 (3.12-3.29) |
|  | eGFR, age | | 1.26 (1.11-1.42) | 1.01 (0.98-1.03) | 0.97 (0.94-0.99) |
|  | Number of events (n) | | 65 | 11081 | 8769 |
| **45-59** (G3a) | Unadjusted | | 0.35 (0.28-0.44) | 1.96 (1.89-2.03) | 2.86 (2.72-3.01) |
|  | eGFR | | 0.36 (0.29-0.45) | 1.81 (1.72-1.90) | 2.35 (2.23-2.48) |
|  | eGFR, age | | 1.22 (0.97-1.54) | 0.89 (0.85-0.93) | 0.92 (0.87-0.98) |
|  | Number of events (n) | | 18 | 4535 | 1643 |
| **30-44** (G3b) | Unadjusted | | 0.38 (0.29-0.51) | 1.74 (1.62-1.86) | **-** |
|  | eGFR | | 0.39 (0.29-0.52) | 1.61 (1.48-1.75) | **-** |
|  | eGFR, age | | 1.09 (0.82-1.45) | 0.99 (0.91-1.08) | **-** |
|  | Number of events (n) | | 12 | 830 | **-** |
| **15-29** (G4) | Unadjusted | | 0.46 (0.37-0.59) | 1.91 (1.28-2.85) | **-** |
|  | eGFR | | 0.45 (0.36-0.57) | 2.11 (1.40-3.18) | **-** |
|  | eGFR, age | | 0.96 (0.75-1.23) | 1.27 (0.84-1.93) | **-** |
|  | Number of events (n) | | 17 | 24 | **-** |
| **<15** (G5) | Unadjusted | | 0.62 (0.48-0.80) | **-** | **-** |
|  | eGFR | | 0.55 (0.42-0.72) | **-** | **-** |
|  | eGFR, age | | 1.01 (0.76-1.34) | **-** | **-** |
|  | Number of events (n) | | 16 | **-** | **-** |

HR = hazard ratio; CI = confidence interval; KFRT = kidney failure with replacement therapy; MACE = major adverse cardiovascular events. See **Supplemental Figure 1** for detailed explanation on this analysis.

Supplemental Table S10. Hazard ratios comparing reclassified and non-reclassified participants across eGFR categories using the CKD-EPI equation, with CKD G3-5 categories merged. Results are shown for three models: unadjusted, adjusted for CKD-EPI eGFR, and adjusted for CKD-EPI eGFR and age. Comparisons include participants reclassified to a lower eGFR category versus those not reclassified, those reclassified downward with a clinically meaningful eGFR difference versus both non-reclassified participants and those reclassified with a small difference, and participants reclassified to a higher eGFR category versus non-reclassified individuals. Cell colors indicate the direction of risk: grey for no difference, orange for increased risk among reclassified participants, and green for reduced risk.

| **G category**  (eGFR in mL/min/1.73m^2^) | | **Model covariates** | **↑ eGFR category** | **↓ eGFR category** | **↓ eGFR category**  (with meaningful difference) |
| --- | --- | --- | --- | --- | --- |
| **KFRT,** HR (95% CI) | | | | | |
| **>90** (G1) | Unadjusted | | **-** | 1.74 (1.41-2.16) | 1.71 (1.35-2.18) |
|  | eGFR | | **-** | 1.38 (1.07-1.79) | 1.35 (1.04-1.76) |
|  | eGFR, age | | **-** | 1.13 (0.87-1.48) | 1.02 (0.77-1.35) |
|  | Number of events (n) | | **-** | 108 | 81 |
| **60-89** (G2) | Unadjusted | | 0.76 (0.38-1.52) | 1.88 (1.42-2.50) | 0.58 (0.3-1.11) |
|  | eGFR | | 1.29 (0.64-2.59) | 0.49 (0.36-0.68) | 0.18 (0.09-0.35) |
|  | eGFR, age | | 1.09 (0.54-2.20) | 0.70 (0.51-0.97) | 0.30 (0.15-0.58) |
|  | Number of events (n) | | 2 | 53 | 9 |
| **<60** (G3-G5) | Unadjusted | | 2.24 (2.02-2.48) | 0.22 (0.16-0.30) | 0.13 (0.05-0.34) |
|  | eGFR | | 1.41 (1.27-1.57) | 0.22 (0.16-0.31) | 0.26 (0.10-0.70) |
|  | eGFR, age | | 0.95 (0.86-1.06) | 0.34 (0.25-0.47) | 0.34 (0.13-0.90) |
|  | Number of events (n) | | 94 | 38 | 4 |
| **All-cause mortality,** HR (95% CI) | | | | | |
| **>90** (G1) | Unadjusted | | **-** | 4.48 (4.40-4.56) | 5.17 (5.08-5.26) |
|  | eGFR | | **-** | 2.47 (2.42-2.53) | 2.97 (2.91-3.03) |
|  | eGFR, age | | **-** | 1.04 (1.02-1.07) | 0.99 (0.96-1.01) |
|  | Number of events (n) | | **-** | 23006 | 20546 |
| **60-89** (G2) | Unadjusted | | 0.25 (0.22-0.29) | 3.73 (3.67-3.78) | 5.42 (5.33-5.51) |
|  | eGFR | | 0.28 (0.24-0.33) | 2.67 (2.62-2.73) | 3.82 (3.74-3.90) |
|  | eGFR, age | | 1.16 (0.99-1.35) | 1.02 (1.00-1.04) | 0.96 (0.94-0.98) |
|  | Number of events (n) | | 41 | 20078 | 16450 |
| **<60** (G3-G5) | Unadjusted | | 0.41 (0.37-0.46) | 1.71 (1.68-1.78) | 2.51 (2.41-2.60) |
|  | eGFR | | 0.38 (0.34-0.42) | 1.53 (1.50-1.57) | 2.59 (2.49-2.69) |
|  | eGFR, age | | 1.17 (1.05-1.30) | 0.97 (0.95-0.99) | 1.01 (0.97-1.05) |
|  | Number of events (n) | | 91 | 9575 | 2950 |
| **MACE,** HR (95% CI) | | | | | |
| **>90** (G1) | Unadjusted | | - | 3.84 (3.77-3.93) | 4.09 (4.00-4.18) |
|  | eGFR | | - | 1.63 (1.59-1.67) | 1.9 (1.86-1.95) |
|  | eGFR, age | | - | 0.74 (0.72-0.77) | 0.68 (0.66-0.70) |
|  | Number of events (n) | | - | 14133 | 11851 |
| **60-89** (G2) | Unadjusted | | 0.40  (0.35-0.45) | 3.40 (3.34-3.47) | 4.69 (4.59-4.80) |
|  | eGFR | | 0.45  (0.40-0.50) | 2.36 (2.30-2.43) | 3.20 (3.12-3.29) |
|  | eGFR, age | | 1.26  (1.11-1.42) | 1.01 (0.98-1.03) | 0.97 (0.94-0.99) |
|  | Number of events (n) | | 65 | 11081 | 8769 |
| **<60** (G3-G5) | Unadjusted | | 0.44 (0.39-0.40) | 1.61 (1.57-1.66) | 2.19 (2.09-2.30) |
|  | eGFR | | 0.42 (0.37-0.47) | 1.46 (1.41-1.50) | 2.26 (2.15-2.38) |
|  | eGFR, age | | 1.09 (0.96-1.24) | 0.97 (0.95-1.00) | 1.00 (0.95-1.05) |
|  | Number of events (n) | | 63 | 5389 | 1643 |

HR = hazard ratio; CI = confidence interval; KFRT = kidney failure with replacement therapy; MACE = major adverse cardiovascular events.

Supplemental Table S11. Event and non-event net reclassification index for study outcomes for the total cohort and within subgroups. 95% confidence intervals were based on 500 bootstrap samples.

|  | **KFRT** | | | **All-cause mortality** | | | **MACE** | | |
| --- | --- | --- | --- | --- | --- | --- | --- | --- | --- |
|  | **Overall NRI^†^ (95% CI)** | **Event NRI, % (95% CI)** | **Nonevent NRI, % (95% CI)** | **Overall NRI^†^ (95% CI)** | **Event NRI, % (95% CI)** | **Nonevent NRI, % (95% CI)** | **Overall NRI (95% CI)** | **Event NRI, % (95% CI)** | **Nonevent NRI, % (95% CI)** |
| **Age ≥ 65 years** | -16.8  (-18.7 to -14.2) | 8.1  (6.23 to 10.7) | -24.9  (-25.0 to -24.7) | 0.8  (0.5 to 1.2) | 25.3  (25.1 to 25.6) | -24.5  (-24.7 to -24.3) | -0.4  (-0.8 to -0.1) | 24.5  (24.2 to 24.8) | -25.0  (-25.2 to -24.8) |
| **Age < 65 years** | -9.4  (-10.9 to -7.8) | -1.1  (-2.6 to 0.5) | -8.3  (-8.3 to -8.2) | 6.7  (6.3 to 7.1) | 14.7  (14.4 to 15.1) | -8.0  (-8.1 to -8.0) | 6.3  (5.9 to 6.7) | 14.4  (14.0 to 14.8) | -8.1  (-8.1 to -8.0) |
| **Female** | -8.0  (-10.3 to -5.7) | 4.3  (2.0 to 6.6) | -12.3  (-12.3 to -12.2) | 13.6  (13.3 to 13.9) | 24.3  (24.0 to 24.6) | -10.8  (-10.8 to -10.7) | 12.4  (12.1 to 12.8) | 23.9  (23.5 to 24.2) | -11.4  (-11.5 to -11.4) |
| **Male** | -9.2  (-10.7 to -7.8) | 0.8  (-0.7 to 2.3) | -10.0  (-10.1 to -10.0) | 10.9  (10.6 to 11.2) | 19.7  (19.4 to 19.9) | -8.7  (-8.8 to -8.7) | 9.1  (8.8 to 9.4) | 18.3  (18.0 to 18.6) | -9.2  (-9.3 to -9.2) |
| **Hypertension yes** | -18.6  (-20.4 to -16.9) | 1.0  (-0.8 to 2.7) | -19.6  (-19.7 to -19.4) | 5.6  (5.2 to 6.0) | 23.4  (23.0 to 23.6) | -17.7  (-17.9 to -17.6) | 3.9  (3.5 to 4.3) | 22.5  (22.1 to 22.8) | -18.6  (-18.8 to -18.4) |
| **Hypertension no** | -7.0  (-8.9 to -5.2) | 3.0  (1.1 to 4.9) | -10.1  (-10.1 to -10.0) | 12.8  (12.6 to 13.1) | 21.8  (21.6 to 22.1) | -9.0  (-9.1 to -9.0) | 10.7  (10.4 to 11.1) | 20.2  (20.0 to 20.5) | -9.5  (-9.6 to -9.5) |
| **Diabetes yes** | -15.6  (-17.8 to -13.6) | 1.7  (-0.6 to 3.6) | -17.3  (-17.6 to -17.1) | 6.1  (5.5 to 6.7) | 21.3  20.8 to 21.8) | -15.2  (-15.5 to -14.9) | 4.6  (4.0 to 5.3) | 20.7  (20.1 to 21.3) | -16.1  (-16.3 to -15.8) |
| **Diabetes no** | -8.9  (-10.5 to -7.2) | 2.0  (0.4 to 3.7) | -10.9  (-11.0 to -10.9 | 12.7  (12.5 to 12.9) | 22.3  (22.1 to 22.5) | -9.6  (-9.7 to -9.6) | 10.8  (-10.5 to 11.1) | 21.0  (10.7 to 21.3) | -10.2  (-10.2 to -10.2) |
| **Cardiovascular disease* yes** | -17.1  (-19.5 to -15.0) | 3.2  (0.9 to 5.2) | -20.4  (-20.4 to -20.0) | 6.9  (6.5 to 7.4) | 24.1  (23.8 to 24.4) | -17.1  (-17.4 to -16.9) | 4.3  (3.8 to 4.7) | 23.0  (22.7 to 23.4) | -18.8  (-19.0 to -18.6) |
| **Cardiovascular disease* no** | -8.8  (-10.3 to -7.3) | 1.5  (0.0 to 3.0) | -10.3  (-10.3 to -10.3) | 11.8  (11.6 to 12.1) | 21.2  (21.0 to 21.5) | -9.4  (-9.4 to -9.3) | 9.8  (-9.9 to -9.8) | 19.7  (19.3 to 20.0) | -9.8  (-10.3 to -7.3) |

KFRT = kidney failure with replacement therapy; MACE = major adverse cardiovascular events; NRI = net reclassification index.

* Cardiovascular disease was defined as a composite of myocardial infarction, ischemic heart disease, heart failure, stroke, other cerebrovascular disease, arrhythmia and peripheral vascular disease.

† Note that the denominators for the event and nonevent NRI are different, e.g. the total number of events is much smaller than the total number of nonevents, which complicates interpretation of the overall NRI. Please see Supplemental Methods for further explanation.

**Supplemental Table 12**. Event and non-event net reclassification index considering only meaningful reclassifications for study outcomes for the total cohort and within subgroups. 95% confidence intervals were based on 500 bootstrap samples.

|  | **KFRT** | | | **All-cause mortality** | | | **MACE** | | |
| --- | --- | --- | --- | --- | --- | --- | --- | --- | --- |
|  | **Overall NRI^†^ (95% CI)** | **Event NRI, % (95% CI)** | **Nonevent NRI, % (95% CI)** | **Overall NRI^†^ (95% CI)** | **Event NRI, % (95% CI)** | **Nonevent NRI, % (95% CI)** | **Overall NRI (95% CI)** | **Event NRI, % (95% CI)** | **Nonevent NRI, % (95% CI)** |
| **Age ≥ 65 years** | -16.2  (-17.5 to -14.9) | 3.7  (2.5 to 5.1) | -20.0  (-19.8 to -20.1) | -0.6  (-0.9 to -0.4) | 19.6  (19.3 to 19.8) | -20.2  (-20.4 to -20.0) | -2.0  (-2.3 to -1.7) | 18.5  (18.2 to 18.7) | -20.4  (-20.6 to -20.3) |
| **Age < 65 years** | -3.4  (-4.1 to -2.5) | 2.4  (-1.7 to 3.3) | -5.8  (-5.8 to -5.7) | 5.1  (4.8 to 5.4) | 10.7  (10.4 to 11.0) | -5.6  (-5.7 to -5.7) | 3.7  (3.4 to 4.0) | 9.4  (9.0 to 9.7) | -5.7  (-5.7 to -5.6) |
| **Female** | -5.6  (-7.0 to -4.0) | 3.9  (2.5 to 5.5) | -9.5  (-9.6 to -9.4) | 11.5  (11.2 to 11.7) | 19.7  (19.3 to 19.8) | -8.2  (-8.3 to -8.2) | 10.0  (9.7 to 10.3) | 18.8  (18.5 to 19.2) | -8.8  (-8.9 to -8.8) |
| **Male** | -4.8  (-5.5 to -4.0) | 2.2  (1.5 to 3.0) | -6.9  (-7.0 to -6.9) | 8.1  (7.9 to 8.4) | 14.1  (13.9 to 14.3) | -6.0  (-6.1 to -6.0) | 5.9  (5.6 to 6.2) | 12.3  (12.1 to 12.6) | -6.4  (-6.5 to -6.4) |
| **Hypertension yes** | -11.9  (-12.6 to -11.0) | 2.0  (1.3 to 2.8) | -13.9  (-14.0 to -13.7) | 4.0  (3.7 to 4.3) | 16.6  (16.3 to 16.9) | -12.6  (-12.7 to -12.4) | 2.4  (2.0 to 2.7) | 15.6  (15.3 to 15.9) | -13.3  (-13.4 to -13.1) |
| **Hypertension no** | -3.9  (-5.0 to -2.9) | 3.7  (2.5 to 5.0) | -7.5  (-7.6 to -7.5) | 10.9  (10.7 to 11.2) | 17.5  (17.3 to 17.9) | -6.6  (-6.7 to -6.6) | 8.3  (8.1 to 8.6) | 15.4  (15.2 to 15.7) | -7.1  (-7.1 to -7.1) |
| **Diabetes yes** | -10.2  (-11.2 to -9.2) | 2.1  (1.1 to 3.1) | -12.3  (-12.5 to -12.1) | 3.8  (3.3 to 4.4) | 14.8  (14.4 to 15.3) | -11.0  (-11.3 to -10.7) | 2.4  (1.9 to 3.0) | 14.0  (13.6 to 14.6) | -11.7  (-11.9 to -11.4) |
| **Diabetes no** | -5.1  (-5.9 to -4.1) | 3.1  (2.2 to 4.0) | -8.1  (-8.1 to -8.1) | 10.4  (10.2 to 10.6) | 17.4  (17.2 to 17.6) | -7.0  (-7.1 to -7.0) | 8.1  (7.9 to 8.4) | 15.7  (15.4 to 15.9) | -7.6  (-7.6 to -7.5) |
| **Cardiovascular disease* yes** | -12.4  (-13.3 to -11.1) | 2.0  (1.0 to 3.2) | -14.3  (-14.5 to -14.2) | 5.2  (4.9 to 5.9) | 17.2  (16.9 to 17.5) | -12.0  (-12.2 to -11.8) | 2.6  (2.3 to 3.0) | 16.1  (15.8 to 16.4) | -13.4  (-13.6 to -13.3) |
| **Cardiovascular disease* no** | -4.6  (-5.5 to -3.7) | 2.0  (1.0 to 3.2) | -14.3  (-14.5 to -14.2) | 10.2  (10.0 to 10.4) | 17.1  (16.9 to 17.3) | -6.9  (-7.0 to -6.9) | 7.7  (7.4 to 8.0) | 15.0  (14.7 to 15.3) | -7.3  (-7.4 to -7.3) |

KFRT = kidney failure with replacement therapy; MACE = major adverse cardiovascular events; NRI = net reclassification index.

* Cardiovascular disease was defined as a composite of myocardial infarction, ischemic heart disease, heart failure, stroke, other cerebrovascular disease, arrhythmia and peripheral vascular disease.

† Note that the denominators for the event and nonevent NRI are different, e.g. the total number of events is much smaller than the total number of nonevents, which complicates interpretation of the overall NRI. Please see Supplemental Methods for further explanation.

Supplemental Figure S1. Flow chart of included participants.


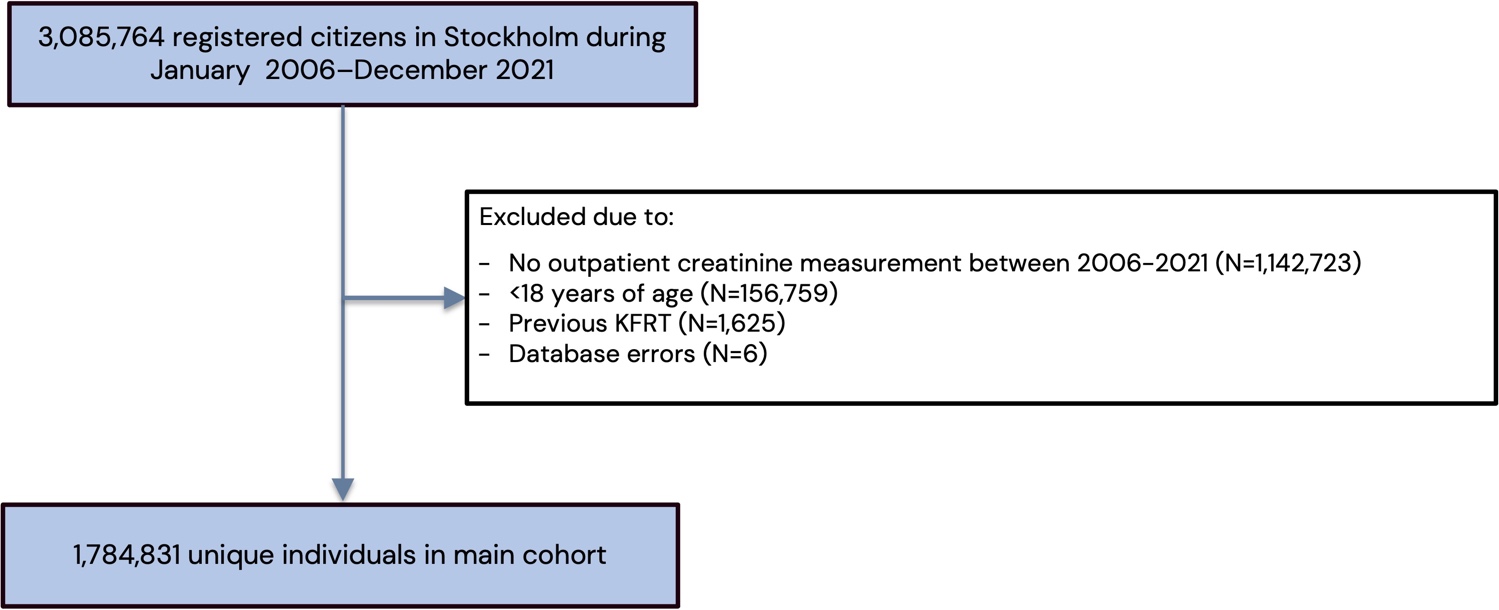


Supplemental Figure S2. Median eGFR across the spectrum of age with CKD-EPI and EKFC equations. Splines are plotted with knots at 30, 40, 50, 60 and 70 years, with 95% confidence intervals plotted as dashed lines.


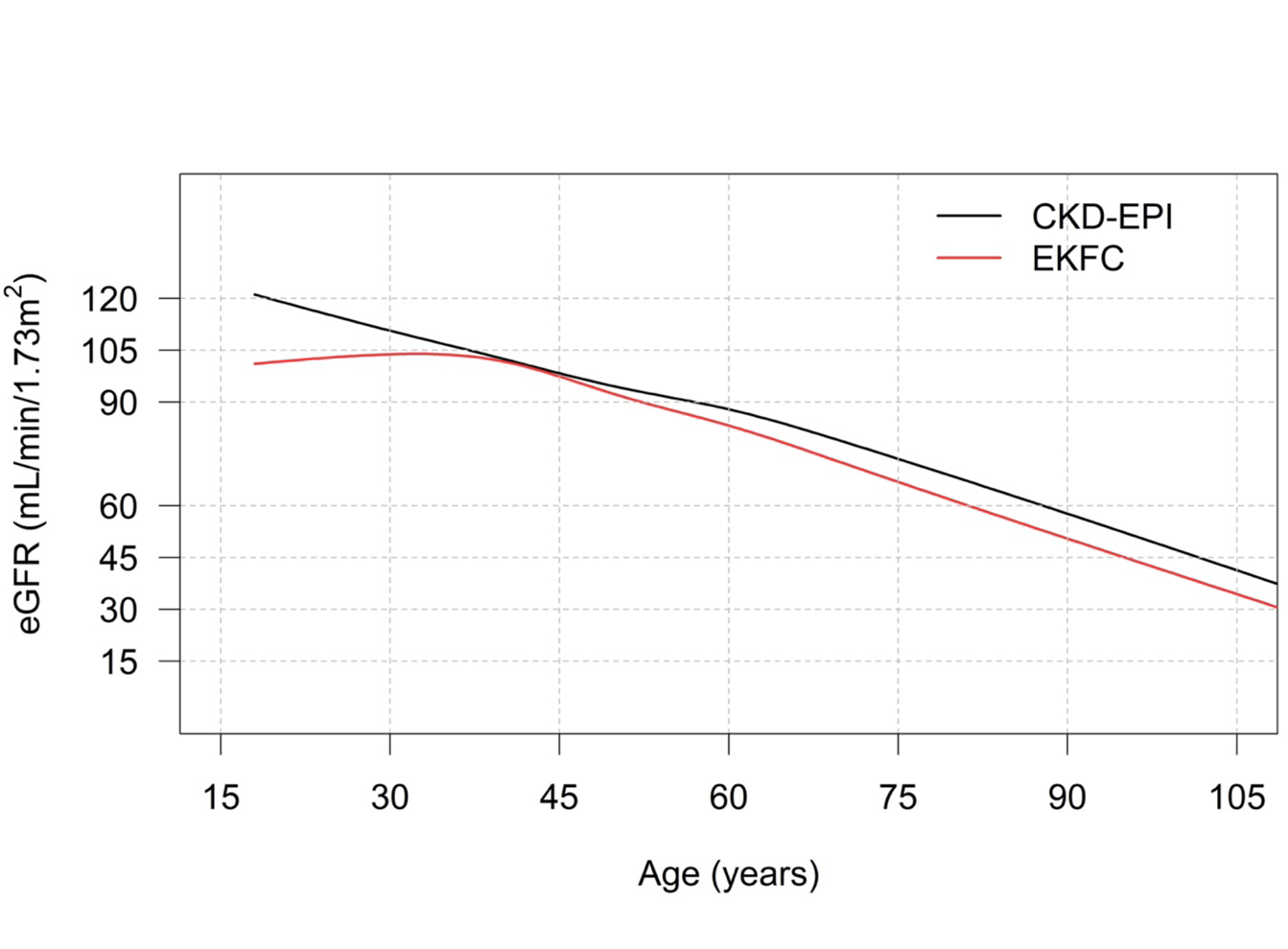


Supplemental Figure S3. eGFR distribution for CKD-EPI and EKFC equations in subgroups of ≥65 vs. <65 years (A-B), ≥40 vs. <40 years (C-D), sex (E-F), hypertension (G-H), diabetes (I-J) and cardiovascular disease (K-L).


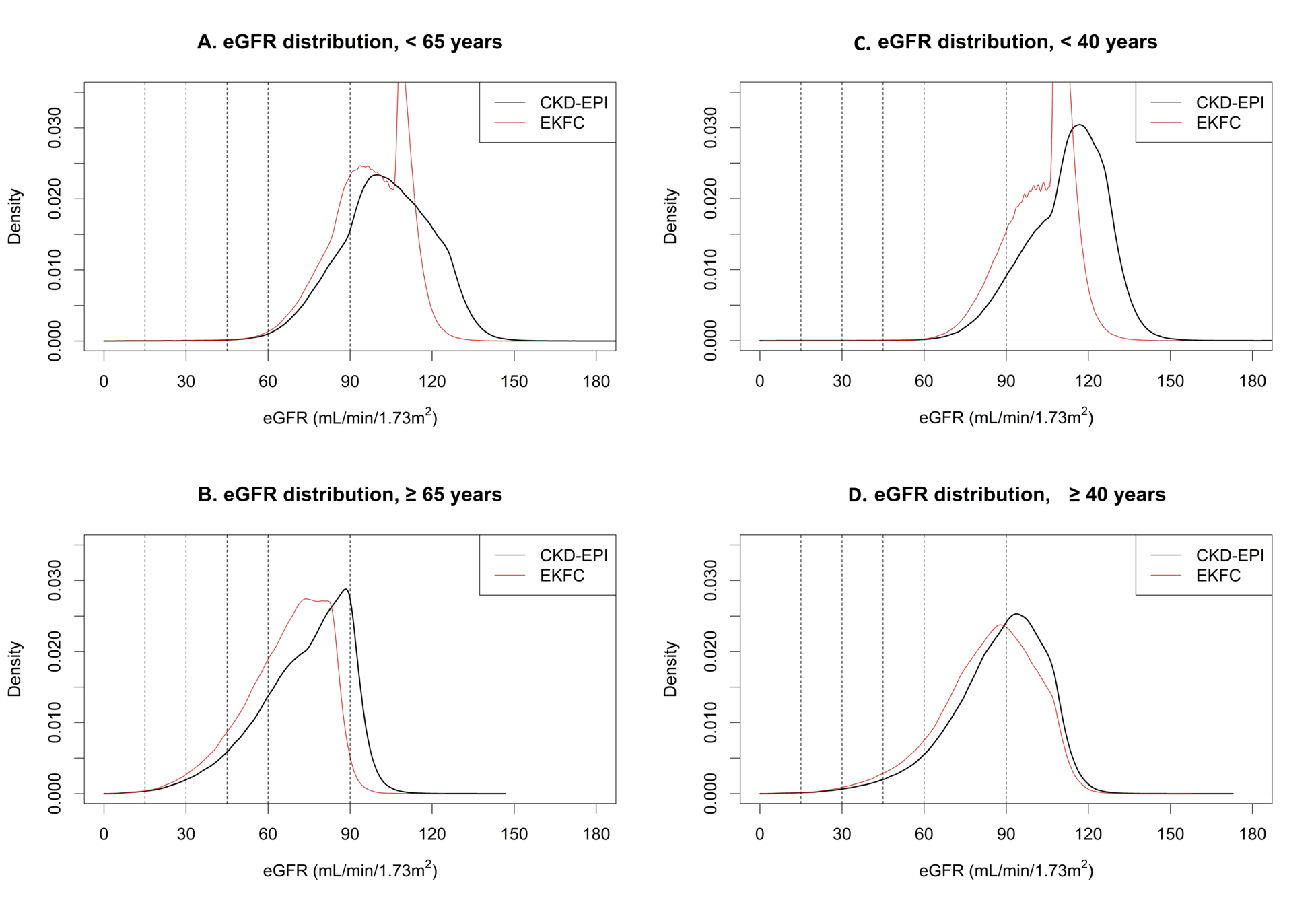


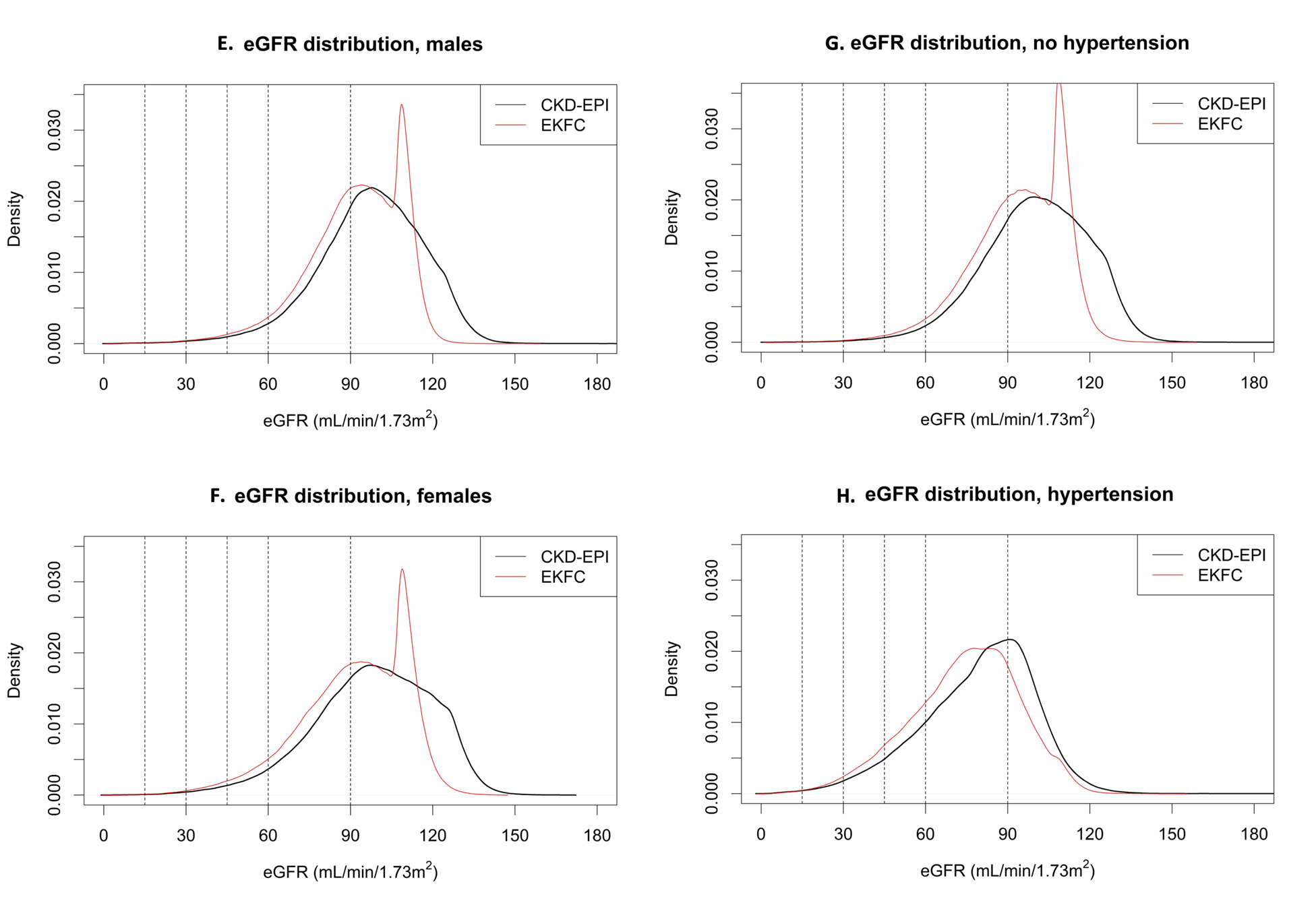


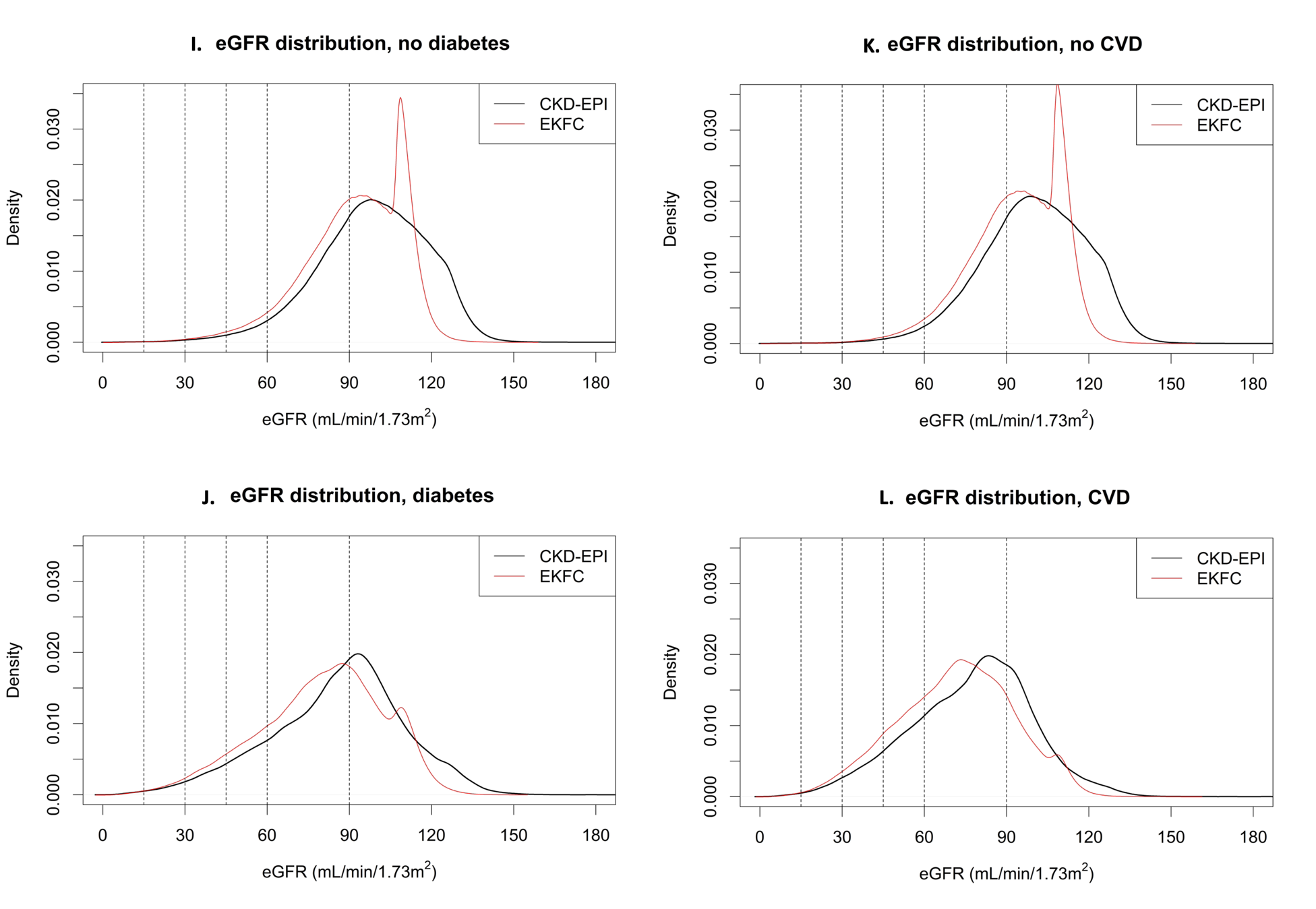


**Supplemental Figure S4**. Hazard ratios for the association between eGFR with the CKD-EPI and EKFC equations and kidney failure with replacement therapy (A), all-cause mortality (B), and major adverse cardiovascular events (C), without adjustment for age, sex and comorbid conditions. An eGFR of 95 mL/min/1.73m^2^ was taken as the reference value. Grey shaded areas depict 95% confidence intervals for the CKD-EPI equation, and dotted red lines depict 95% confidence intervals for the EKFC equation. eGFR was modelled as a restricted cubic spline with five knots at the 5^th^, 27.5^th^, 50^th^, 72.5^th^, and 95^th^ percentile. Note that the Y-axis is on a log-scale.


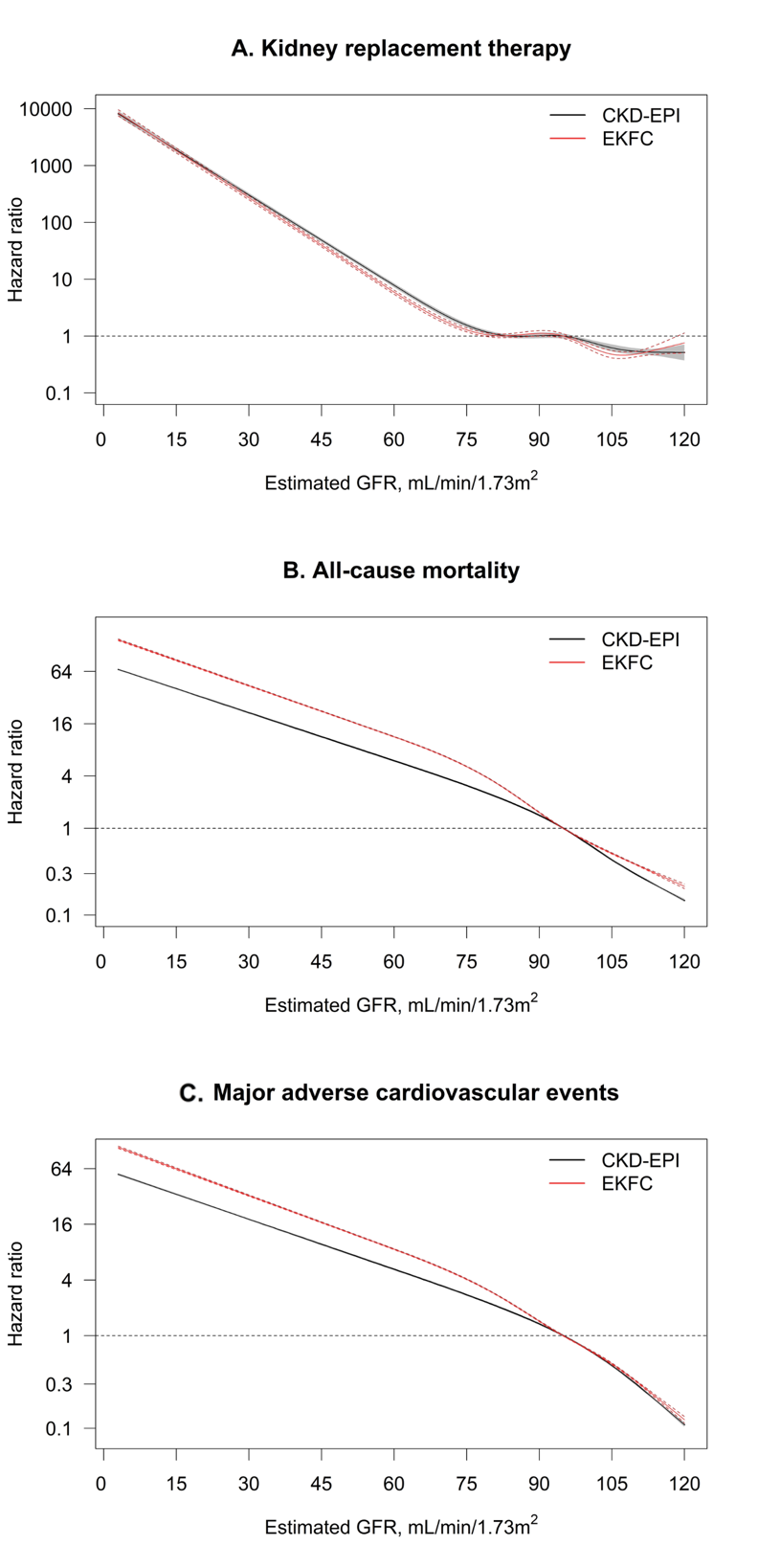


Supplemental figure S5. Hazard ratios for the association between eGFR with CKD-EPI and EKFC equations and kidney failure with replacement therapy (A), all-cause mortality (B), and major adverse cardiovascular events (C) adjusted for age and sex. Age was modelled linearly in the Cox model and an eGFR of 95 mL/min/1.73m^2^ was taken as the reference value.


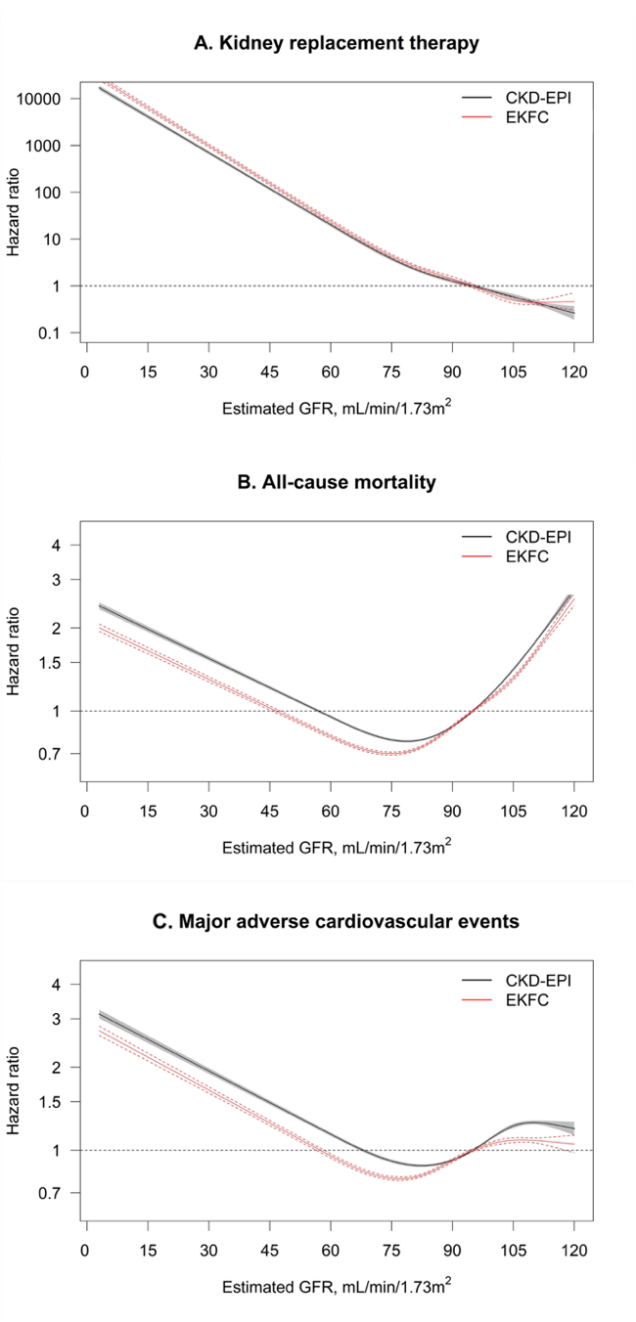


Supplemental Figure S6. Hazard ratios for the association between eGFR with the CKD-EPI and EKFC equations and kidney failure with replacement therapy (A), all-cause mortality (B), and major adverse cardiovascular events (C), without adjustment for age, sex and comorbid conditions. An eGFR of 85 mL/min/1.73m^2^ was taken as the reference value.


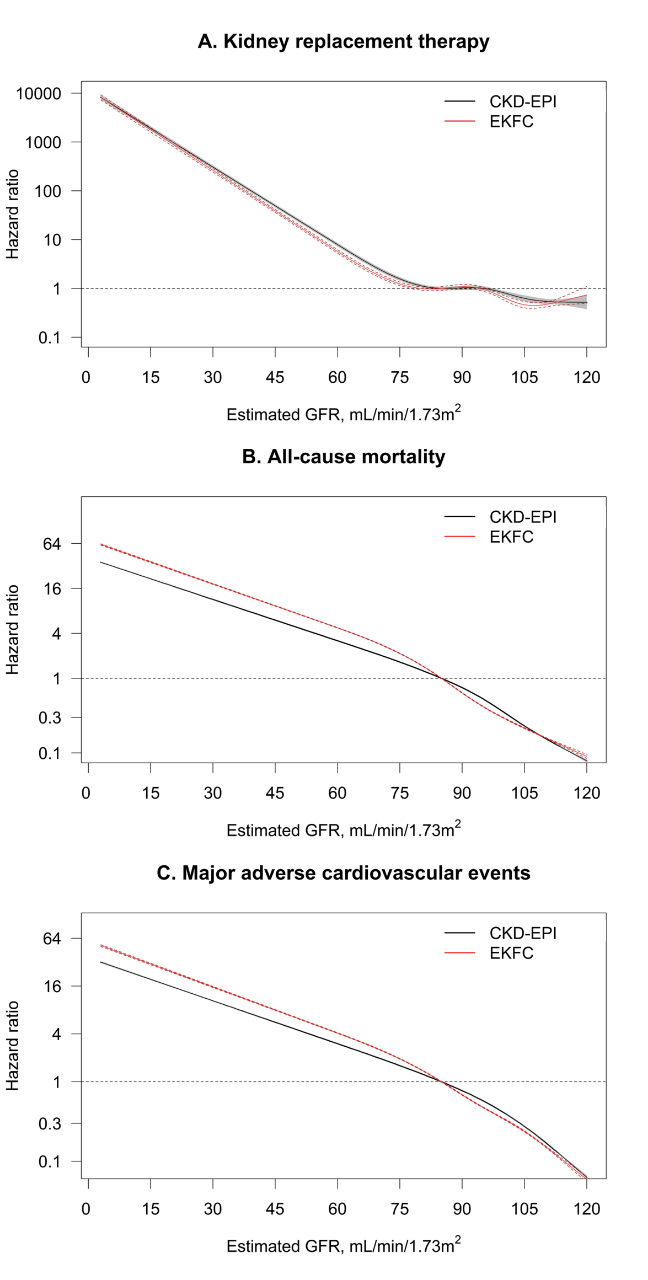


Supplemental Figure S7. Hazard ratios for the association between eGFR with CKD-EPI and EKFC equations and kidney failure with replacement therapy (A), all-cause mortality (B), and major adverse cardiovascular events (C) adjusted for age and sex using an eGFR reference value of 85 ml/min/1.73m^2^. Age was modelled linearly in the Cox model.


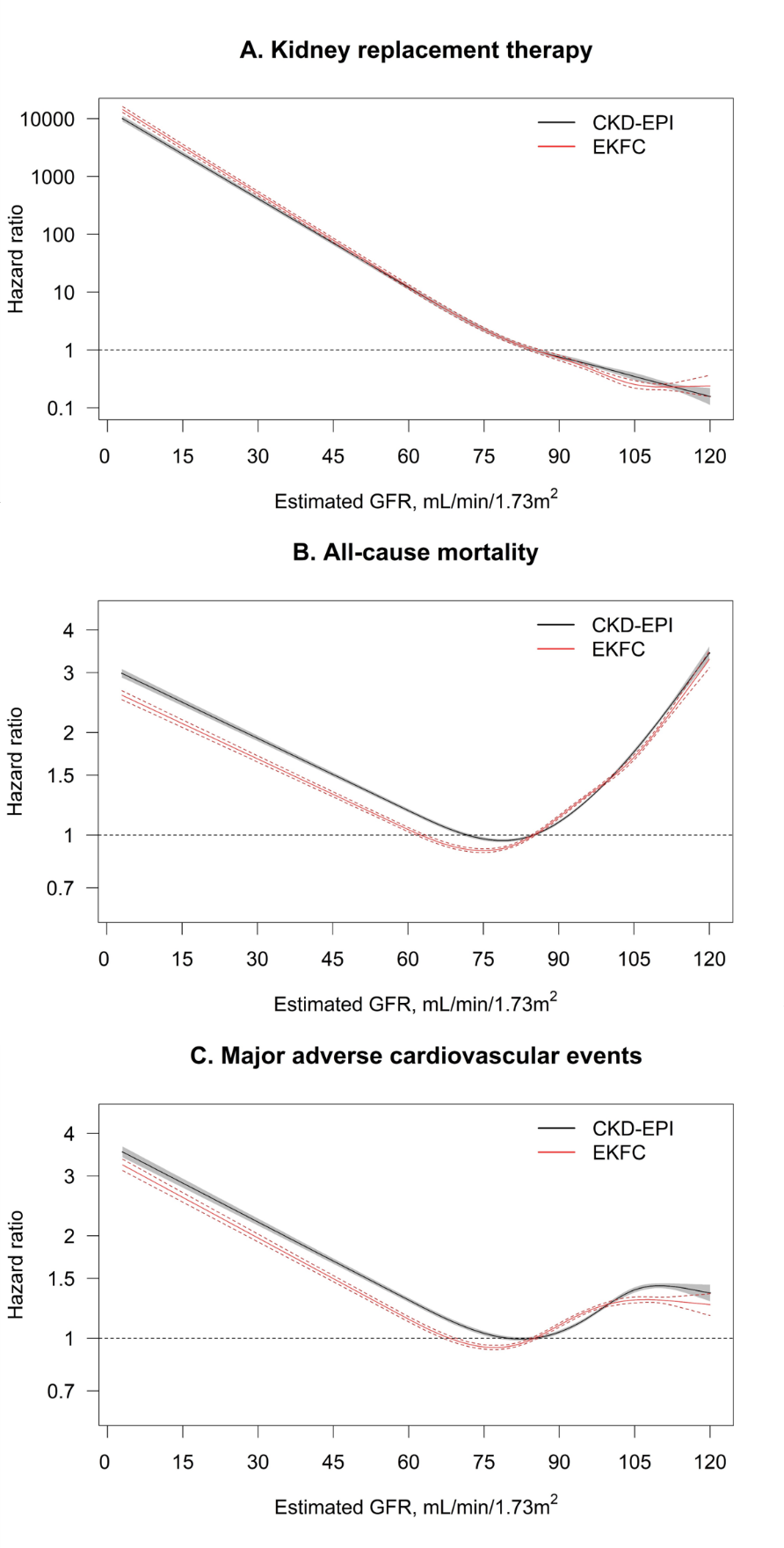


Supplemental Figure S8. Hazard ratios for the association between eGFR with CKD-EPI and EKFC equations and kidney failure with replacement therapy (A), all-cause mortality (B), and major adverse cardiovascular events (C) adjusted for age, sex, comorbidities and medication use with a reference eGFR value of 85 ml/min/1.73m^2^. Age was modelled linearly in the Cox model. Hazard ratios were adjusted for comorbidities (hypertension, myocardial infarction, ischemic heart disease, heart failure, stroke, cerebrovascular disease, arrhythmia, peripheral vascular disease, diabetes mellitus, cancer, chronic obstructive disease and liver disease) and medication use (β-blockers, calcium channel blockers, diuretics, renin-angiotensin system inhibitors, lipid-lowering drugs and non-steroidal anti-inflammatory drugs.


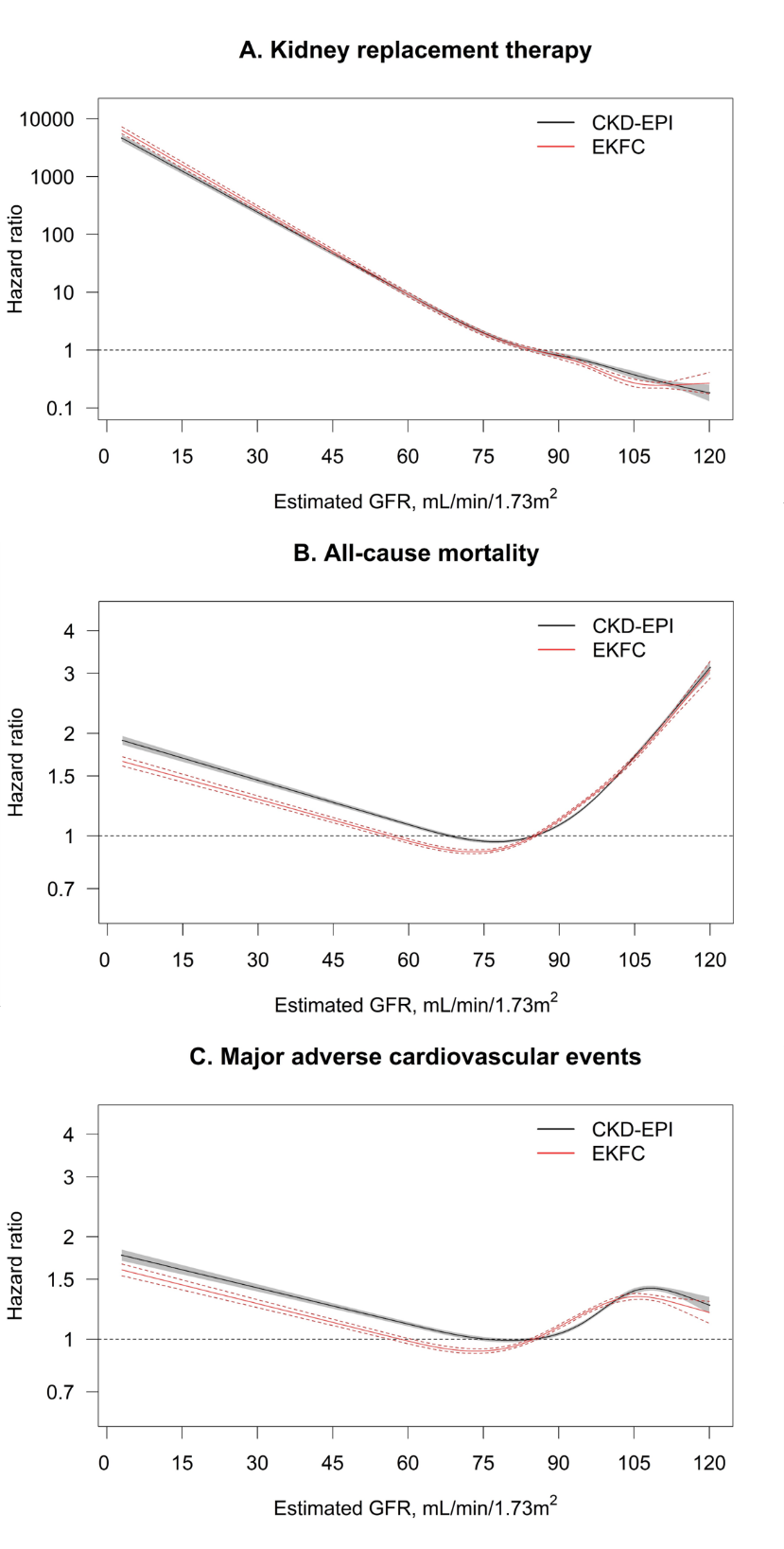

Supplement: gfaf148_Supplemental_File [file gfaf148_Supplemental_File.docx]
